# Spontaneous water-on-water spreading of polyelectrolyte membranes inspired by skin formation

**DOI:** 10.1038/s41467-022-30973-6

**Published:** 2022-06-09

**Authors:** Sihan Tang, Jiang Gong, Yunsong Shi, Shifeng Wen, Qiang Zhao

**Affiliations:** 1 Key Laboratory of Material Chemistry for Energy Conversion and Storage (Ministry of Education), School of Chemistry and Chemical Engineering, Huazhong University of Science and Technology, Wuhan, 430074 P. R. China; 2 State Key Laboratory of Materials Processing and Die & Mould Technology, Huazhong University of Science and Technology, Luoyu Road No. 1037, 430074 Wuhan, China; 3 Department of Orthopaedics, Union Hospital, Tongji Medical College, Huazhong University of Science and Technology, 430022 Wuhan, China

**Keywords:** Polymers, Surface assembly, Polymers

## Abstract

Stable interfaces between immiscible solvents are crucial for chemical synthesis and assembly, but interfaces between miscible solvents have been less explored. Here the authors report the spontaneous water-on-water spreading and self-assembly of polyelectrolyte membranes. An aqueous mixture solution containing poly(ethyleneimine) and poly(sodium 4-styrenesulfonate) spreads efficiently on acidic water, leading to the formation of hierarchically porous membranes. The reduced surface tension of the polyelectrolyte mixture solution drives the surface spreading, while the interfacial polyelectrolytes complexation triggered by the low pH of water mitigates water-in-water mixing. The synergy of surface tension and pH-dependent complexation represents a generic mechanism governing interfaces between miscible solvents for materials engineering, without the need for surfactants or sophisticated equipment. As a proof-of-concept, porous polyelectrolyte hybrid membranes are prepared by surface spreading, exhibiting exceptional solar thermal evaporation performance (2.8 kg/m^2^h) under 1-sun irradiation.

## Abstract (web summary)

Stable interfaces between immiscible solvents are crucial for chemical synthesis and assembly, but interfaces between miscible solvents have been less explored. Here the authors report the spontaneous water-on-water spreading and self-assembly of polyelectrolytes.

## Introduction

Interfaces between immiscible liquids play crucial roles in chemistry and engineering, such as emulsions^1–4^, reactions^5–7^, nanofabrication^8–10^, fluidics^11–13^ and more. A classic phenomenon is the oil-on-water spreading^14^, which dates back centuries and underpins cutting edge materials including defined monolayers, thin films, and superstructures^15–18^. In these scenarios, water-immiscible dispersing solvents with smaller surface tension and lower density than water are used. By contrast, water or water-miscible solvents normally mix in water quickly, hindering the formation of stable interfaces and occurrence of solvent-on-water spreading^14^. The solvent-in-solvent mixing could be circumvented in part, when the size of a solvent droplet was reduced by many orders of magnitude (i.e., from millimeters to microns), through instruments such as high-voltage electrospray^19^. By this method, hydrophobic particles and amphiphilic nanosheets dispersed in water-miscible solvents were assembled on water, indicating the significant potential of interfaces between miscible solvents^20–23^. However, both the tinny sizes of solvent droplets and the use of high voltage (10 kV) technologies hinder its productivity. Moreover, the spreading of water-soluble polymers on water is more challenging as they could dissolve and diffuse in water. Collectively, principles of manipulating water-on-water spreading are needed.

When it comes to biology, water is the only solvent in organisms, with little-to-no organic solvents involved. Million years of evolution has endowed organisms with the intriguing ability to manipulate water ~ water interfaces for intracellular compartments^24–26^ and biological materials spanning multiple length scales^27–30^. A recent report shows that the pH-responsive liquid–liquid phase separation of protein mixtures drives the formation of skin barriers^30^. In brief, fluidic Keratohyalin granules undergo pH-responsive phase changes during their upward journey toward the skin surface, ending up with the liquid spreading and formation of squame^30^. The water ~ water interfaces in granules were modulated by pH-responsive interactions between proteins, which hints that stimuli-dependent interactions between water-soluble polymers might be viable for stabilizing water ~ water dynamic interfaces between two aqueous polymer solution. With this inspiration, we consider polyelectrolytes as model polymers to mimic proteins as they bear charges that enable stimuli-responsive polyelectrolyte complexation analogous to protein complexation^31–33^. It is speculated that stimuli-responsive phase changes of polyelectrolytes solution would be viable to actuate the water ~ water interfaces and water-on-water spreading.

Here, we report a skin-inspired strategy to accomplish spontaneous, water-on-water (SWOW) spreading of polyelectrolytes. The key to design SWOW is the coupling of pH-dependent polyelectrolyte complexation to water ~ water interfaces. A mixture solution containing polyethylenimine (PEI) and sodium polystyrene sulfonate (PSSNa) was prepared. In this design, the PSSNa endows the mixture solution with surface tension lower than water, which is the driving force for surface spreading. Meanwhile, PEI is a weak polyelectrolyte bearing positive charge at acid condition, which renders pH-responsive complexation^34–36^. Droplets of PEI-PSSNa solution spontaneously spread on acidic water, forming hierarchical porous membranes. The SWOW spreading is conveniently operated using a pipette at ambient conditions, without the need for miniaturized droplets and complicated technologies. It applies to various polyelectrolytes and nanomaterials, and expands the scope of functional materials accessible by surface spreading. As a proof-of-concept, these porous membranes show good solar thermal evaporation performance under 1-sun irradiation.

## Results

### Skin-inspired design of SWOW spreading systems

**Fig. 1:**
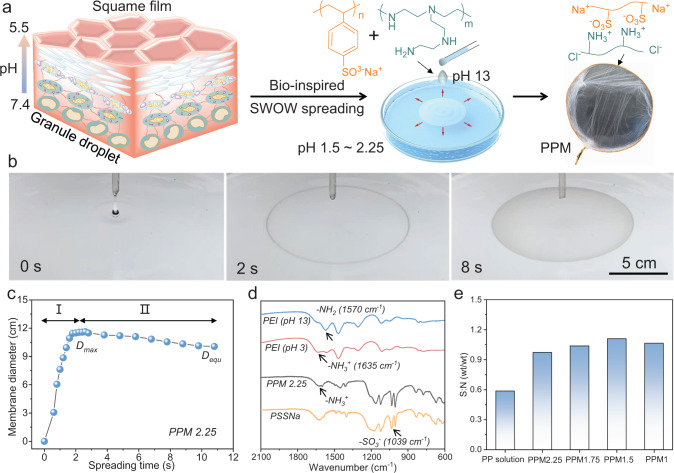
Bio-inspired preparation of PPMs by SWOW spreading. **a** Schematic illustration of the formation of skin barriers (left): near the skin surface, the change of intracellular pH value (from 7.4 to 5.5) drives phase separation to form squama. This process inspires the coupling of pH-responsive PEI-PSSNa complexation to water–water interface (right). **b** Optical photographs of a drop (~40 μL) of PEI-PSSNa solution spreading on water (pH 2.25, 25 ^o^C). **c** Effect of spreading time on the diameter of PPM2.25. **d** FT-IR curves of PPM2.25 and PEI. **e** Elemental analysis of PPMx (x: 2.25, 1.75, 1.5, 1). Note: PP means PEI-PSSNa solution.

As shown in Fig. 1a, the fluidic protein granules undergo spreading and solidification actuated by the pH decrease (7.4–5.5) and concurrent protein complexation during their upward journey toward the skin surface^30^. In this process, water ~ water dynamic interfaces were stabilized by pH-dependent protein complexation (Supplementary Fig. 1). This principle leads us to consider that the pH-dependent charge of week polyelectrolyte, which is known to the field of polyelectrolytes and membranes^34–36^, is a versatile tool to stabilize water ~ water dynamic interfaces for SWOW spreading. Two commercial polyelectrolytes, i.e., PEI and PSSNa, were dissolved in water (pH 13) to prepare a homogenous mixture (Supplementary Fig. 2). The protonation of amine groups in PEI is low at pH 13, leading to weak PEI-PSSNa complexation. The principle to prepare homogeneous polyelectrolytes mixture is to first screen the charge attraction between different polyelectrolytes, which is consistent with previous reports^37–39^.

Figure 1b shows that a droplet of PEI-PSSNa solution spontaneously spreads on water (pH 2.25) surface, forming a PEI-PSSNa Membrane (PPMx, x denotes the pH of water) in ~2 s. The diameter (*D*) of PPM2.25 linearly depends on the spreading time in the beginning (stage I, Fig. 1c), after which the membrane gradually contracts (>2 s, stage II). The maximum (*D*_max_) and equilibrium (*D*_equ_) diameters of PPM2.25 are ~12 cm and ~10 cm, respectively, which also depend on the volume of the droplet (Supplementary Fig. 3). Finally, the water-floating PPM2.25 was brought in air as a free-standing membrane (Fig. 1a). In control experiments, both the PSSNa and PEI aqueous solution dissolve in pH 2.25 water, without the occurrence of SWOW phenomenon. In literature, water ~ water interfaces can exist in the bulk of two-phases aqueous systems^21^, which are not viable for water-on-water surface spreading.

Figure 1d shows that PEI (pH 13) reveals a characteristic FT-IR peak at 1570 cm^−1^ (blue arrow), which is assigned to amine (-NH_2_) groups^40^. When the pH of PEI was tuned to 3, the 1570 cm^−1^ peak shifts to 1635 cm^−1^ (red arrow), which is due to the protonated amine (-NH_3_^+^) groups in PEI. Similarly, the PPM2.25 shows peaks at both 1635 cm^−1^ and 1570 cm^−1^, indicating that amine groups in PPM2.25 were protonated during the SWOW spreading of PEI-PSSNa solution on acidic water (pH 2.25). In addition, PPM2.25 also shows the characteristic peak (1039 cm^−1^) for sulfonate groups from PSSNa. Figure 1e shows that the mass ratio of sulfur and nitrogen elements (S:N) in different PPMx (*x* = 2.25, 1.75, 1) is stable around 1. Finally, the content of Na^+^ in the PPM2.25 is very low (~0.1 wt%), while the Na^+^ content in PSSNa is calculated to be ~12 wt%. This is a direct proof of the PEI-PSSNa electrostatic complexation, during which the Na^+^ counter ions of PSSNa were released as free ions and removed during the membrane washing (Supplementary Fig. 4).

**Fig. 2:**
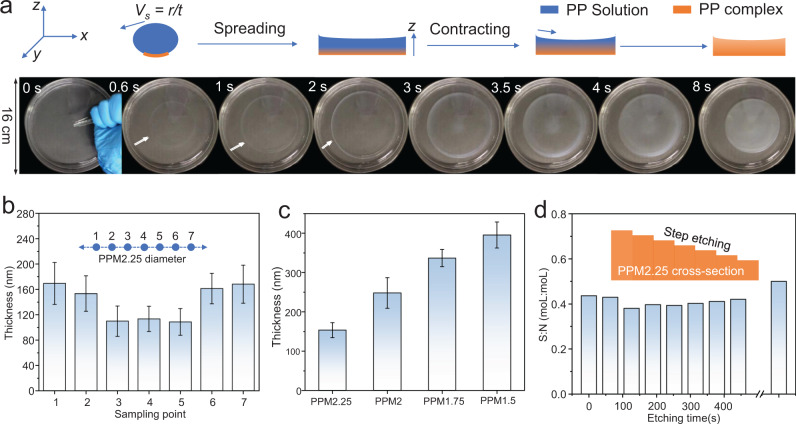
Thickness and composition characterizations of PPM2.25. **a** Optical photographs of a PEI-PSSNa droplet spreading on pH 2.25 water; inner diameter of the glass vessel is 16 cm. Schemes are inserted to illustrate the cross-sectional changes of the droplet. **b** Thickness of PPM2.25 at seven different locations along the diameter direction. **c** Average thickness of PPMs prepared by SWOW on water with different pH. **d** Mole ratio of sulfur and nitrogen elements at different depth down the PPM2.25 cross-section. Error bars: standard deviation. Note: error bars in (**b**, **c**) are standard deviations. PP solution: PEI-PSSNa solution.

The contraction of PPM2.25 was further studied. As shown by arrows in Fig. 2a, the expanding periphery of PPM2.25 turns opaque quickly in the first 2 s of droplet spreading, while the rest of PPM2.25 remains transparent (Supplementary Movie 1). This opaque periphery represents outer borderline of the spreading droplet, whereby the PEI-PSSNa complexation occurs since the beginning of the spreading process. With increasing the spreading time, this periphery becomes more and more solidified, which has a self-limiting effect in the droplet spreading. That is, the spreading solution eventually bounces back against the solidified periphery, resulting in the backflow of PEI-PSSNa solution accompanied with the contraction of PPM2.25. Meanwhile, as indicated by the change of orange color of the cross-sectional schemes in Fig. 2a, the complexation at thickness (*z*-axis) direction started from the bottom surface contacting with acidic water, and progressed toward the membrane’s top surface contacting with air. The PEI-PSSNa complexation would result in phase separation, which was verified by the membrane’s increasing opaqueness with increasing time (>3 s).

Thickness of air-dried PPM2.25 was measured along the diameter direction. Figure 2b shows that the PPM2.25 is thicker (~160 nm) at its periphery part, thinner at the central part (~120 nm). This thickness profile is consistent with the membrane contraction of PPM2.25, i.e., the backflow of PEI-PSSNa solution renders higher thickness at the membrane periphery. Figure 2c shows that the average thickness of PPMs decreases with increasing pH of water. The PEI-PSS complexation is stronger at lower pH, leading to smaller *D*_max_ and higher thickness of PPMs. As shown by the inserted scheme in Fig. 2d, PPM2.25 was etched stepwise using argon-ions and measured by XPS. The mole ratio of sulfur element to nitrogen element is stable (~0.45) at different depth down the PPM2.25, and the sodium content throughout the membrane cross-section is very low (~0.1 wt%). These results suggest that both the complexation and chemical composition at thickness direction are homogeneous.

**Fig. 3:**
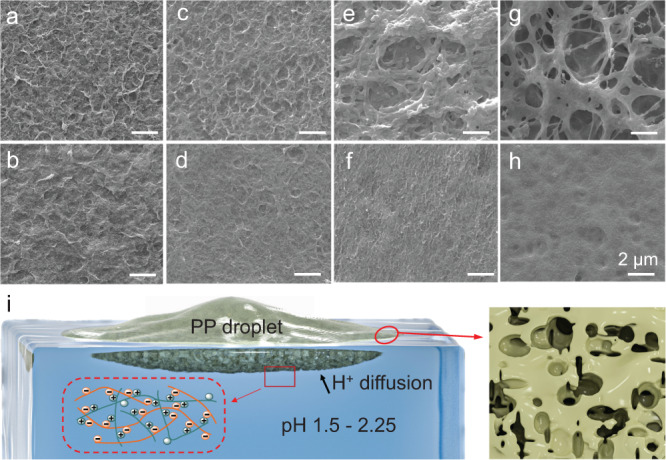
Structure characterizations of PPMs. Cryo-SEM images of (**a**, **b**) PPM2.25, (**c**, **d**) PPM2, (**e**, **f**) PPM1.75, and (**g**, **h**) PPM1.5. All scale bars are 2 μm. Images in (**a**, **c**, **e**, **g**), and (**b**, **d**, **f**, **h**) are top (contacting with air) and bottom (contacting with water) surfaces of PPMs, respectively. **i** A scheme illustrating the SWOW spreading and structures of PEI-PSSNa solution. Note: PP droplet means PEI-PSSNa solution droplet.

Figure 3 shows the Cryo-SEM characterizations of PPMs. As seen from Fig. 3 (top row), top surfaces (contacting with air) of all PPMs are rougher and more porous compared to bottom surfaces (contacting with water) which are smoother and less porous (middle row). The asymmetric pore structures of PPMs are consistent with membranes prepared by non-solvents induced phase separation^41^ or aqueous phase separation^42^, which consist of denser skin layer contacting with non-solvents (analogs to water in this work) are denser. Moreover, pore sizes of PPMs increase with decreasing the pH of water. The PEI-PSS complexation is weaker and the spreading is quicker at higher pH, accompanied with membrane contraction as discussed before. In this scenario, the complexation is not strong enough to effectively solidify phase separated pores, and the backflow of PEI-PSSNa solution during the membrane contraction will fill in the pores formed during the contraction. As such, pore structures in PPMx (*x* = 2.25 and 2.0) are less developed. Noteworthy, the creation of asymmetric pores in water-soluble polymers is more challenging compared to hydrophobic polymers^43^.

As schemed in insertion of Fig. 3i, amine groups in PEI were protonated by acidic water, as supported by FT-IR characterizations (Fig. 1d) and the low Na^+^ content in PPMs. Consequently, the protonated PEI undergo concurrent PEI-PSSNa complexation that occurs rapidly^36^. The complexation yields a thin layer of water-insoluble polyelectrolyte complexes (PEC) that stabilizes transient water ~ water interfaces^44^. The complexation gradually progresses upward toward with increasing time, leading to phase inversion and pores^42^. In literature, polyelectrolyte complexation mainly occurs in liquid–liquid^36–38^, or liquid–solid interfaces^45,46^. Here, PEI-PSS complexation is coupled to the surface spreading of aqueous solution on water.

### Mechanism of SWOW spreading

**Fig. 4:**
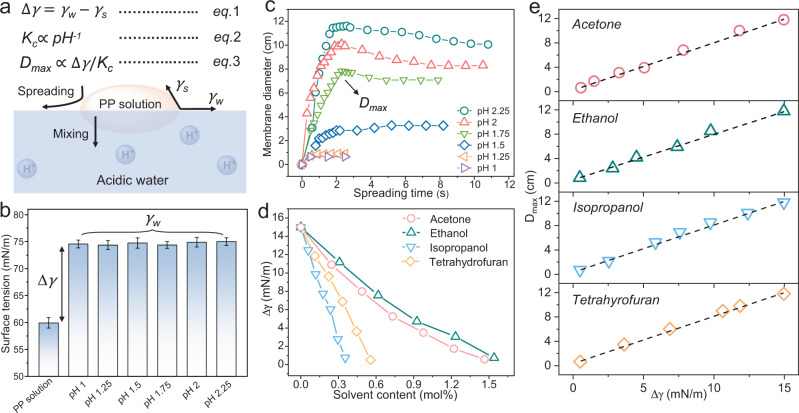
Verification of the SWOW mechanism. **a** A schematic illustration of the effect of surface tension difference (Δ_*γ*_) and PEI-PSSNa complexation (*K*_c_) on maximum diameter (*D*_max_) of the PPMs. **b** Surface tension of PSSNa-PEI solution, and water (pH: 1, 1.25, 1.5, 1.75, 2, 2.25). Error bars: standard deviation. **c** Dependence of the of PPMs diameter on time when PEI-PSSNa solution was placed on water with different pH. **d** Dependence of Δ*γ* on the amount of organic solvents added in water. **e** Dependence of *D*_max_ of PPM2.25 on Δ*γ*. Note: Δ_**γ**_ = *γ*_*w*_ − *γ*_*s*._ Note: PP solution means PEI-PSSNa solution.

As schemed in Fig. 4a, three processes would occur when a drop of PEI-PSSNa solution was placed on acidic water: (1) the surface spreading driven by surface tension, (2) the intrinsic water-in-water mixing, and (3) the pH-dependent PEI-PSSNa complexation. The surface tension difference (Δ*γ*) between water (*γ*_w_) and PEI-PSSNa solution (*γ*_s_) represents the driving force of SWOW spreading (Eq. 1). Noteworthy, the value of *γ*_w_ would vary slightly during the spreading of PEI-PSSNa solution, and the *γ*_w_ is measured to be 74 ± 0.5 mN/m (>*γ*_s_) when the spreading is finished. Here, the static *γ*_w_, instead of dynamic *γ*_w_, was used to qualitatively analyze the effect of Δ*γ* in the maximum diameter of PPMs (Eq. 1). As discussed above, the PEI-PSSNa interfacial complexation was triggered by the lower pH of underlying water. The complexation (K_c_) is stronger when the pH of water is lower (Eq. 2), because the protonation of PEI is positively correlated to acidity of underlying water. The PEI-PSSNa complexation leads to solution-to-solid transition that self-limits both the solution spreading and mixing. As such, the maximum diameter of PPMs (*D*_max_) is positively proportional to Δ*γ* and inversely proportional to K_c_, i.e., *D*_max_ ∝ Δ*γ*/*K*_c_, (Eq. 3). According to Eq. (3), validity of SWOW mechanism was verified by modulating *D*_max_ of PPMs through Δ*γ* (Fig. 4b, c) and K_c_ (Fig. 4d, e) parameters.

Figure 4b shows that *γ*_s_ is 60 mN/m (Supplementary Fig. 5), and the *γ*_w_ is stable around 75 mN/m despite varied pH of water, thus the Δ*γ* is stable (15 mN/m) versus pH of water. On the other hand, the K_c_ is inversely correlated to pH of water (Eq. 2, Fig. 3a), i.e., the PEI-PSSNa complexation is stronger when the pH of water is lower. According to Eq. 3, the *D*_max_ should be positively correlated to pH values of water. Indeed, Fig. 4c shows that the *D*_max_ of PPMs rises from 1 cm to 12 cm with increasing water pH from 1 to 2.25, whereby the hydrophilicity of PPMs enhances with increasing pH (Supplementary Fig. 6). With further increasing the pH of water (pH > 2.5), the PEI-PSSNa complexation (*K*_c_) is too weak to trigger liquid–solid phase transition, and no solid membrane was formed (Supplementary Fig. 7). With decreasing solution pH below 1.25, the complexation (*K*_c_) is so strong that the PEI-PSSNa solution droplet was instantly solidified on surface of acidic water, i.e., very small *D*_max_ (Fig. 4c, triangles).

Figure 4d shows that the value of Δ*γ* decreases linearly when a small amount (0 ~ 2 mol%) of organic solvents (tetrahydrofuran, ethanol, isopropanol and acetone) were added to water, because of the lower surface tension of these solvents (20–28 mN/m). Meanwhile, pH of water was maintained stable (pH 2.25) versus adding organic solvents (Supplementary Fig. 8). When the PEI-PSSNa droplet was placed on surface of water-organic mixture, the *D*_max_ of PPM2.25 decreases linearly with decreasing Δ*γ* (Fig. 4e), that is, *D*_max_ decreases with increasing the amount of solvents added. The linearity of *D*_max_ ~ Δ*γ* lines in Fig. 4e, despite of the varied types of solvents added, indicates the validity of the speculated mechanism (i.e., Eq. 3). In addition, similar *D*_max_ ~ *Δγ* correlation was observed when *γ*_w_ was tuned by adding surfactants in water (Supplementary Fig. 9). The effective prediction and modulation of *D*_max_ by Eq. 3 indicate the validity of SWOW mechanism.

### Broad utility of the SWOW spreading

**Fig. 5:**
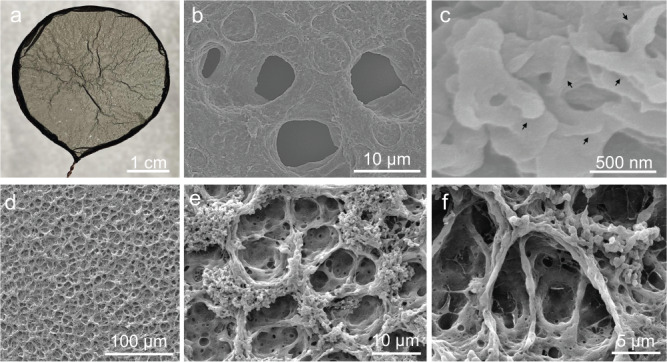
PEI-PSSNa-CNT membranes prepared by SWOW. **a** An optical photograph, (**b**, **c**) SEM and (**d**–**f**) Cryo-SEM photographs of the PPCM1.5. Note: the CNT content in the hybrid membrane is 3 wt%.

The SWOW mechanism applies to different stimuli-responsive polyelectrolyte complexation systems triggered by salts (Supplementary Fig. 10) and solvent exchange (Supplementary Fig. 11). The SWOW mechanism is also viable for fabricating PEC hybrid membranes. A small amount of carbon nanotubes (CNTs) were dispersed in PEI-PSSNa solution, which was spread on acidic water to form PEI-PSSNa-CNT hybrid membranes (PPCMs) that were characterized by Cryo-SEM (Supplementary Fig. 12). PPCM1.5 with relatively larger thickness was chosen for photothermal evaporation study. PPCM1.5 appears black (Fig. 5a), and is stable in water and common organic solvents (Supplementary Fig. 13). Compared to multilayered PEC membranes which involve tens of cycles deposition^46^, the SWOW preparation of PEC films is facile. SEM examination of the freezing-dried PPCM1.5 shows large pores (Fig. 5b) and fine dispersion of CNTs, consistent with optical micrographs characterizations (Supplementary Fig. 14). As indicated by arrows in Fig. 5c, CNTs were tightly embedded in the PEI-PSS matrix. It is worth mentioning that zeta potential of CNTs dispersion (0.1 mg/L) is −10 mV, indicating that CNTs bear moderate negative charge which strengthens their interaction with PEI polymers. Cryo-SEM characterizations (Fig. 5d–f) verify the porous structures in PPCM1.5, which are analogs to pores of PPMs. It is speculated that the added CNTs could serve as rigid fillers which help to reserve the pores during the SWOW and solution-to-solid phase transition. Compared to PPMs, the addition of moderate amount of CNTs does not significantly change the SWOW spreading of PEI-PSSNa-CNT mixture, in terms of spreading speed, membrane diameter, and pore structures (Supplementary Fig. 15). The high porosity of PPCM1.5 is beneficial to solar-thermal applications^47^.

**Fig. 6:**
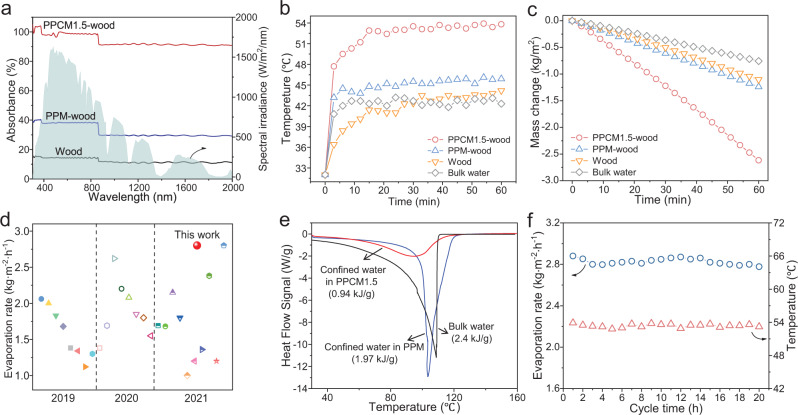
Photothermal evaporation performance of PPCM1.5. **a** UV-vis-NIR absorption spectra of PPCM1.5-wood, PPM-wood, and wood. **b** Surface temperature and (**c**) evaporation rate of PPCM1.5-wood, PPM-wood, wood and bulk water under 1 kW/m^2^ irradiation. **d** Comparison of the evaporation rate of PPCM1.5-wood with some previously reported photothermal materials. **e** DSC curves of confined water in PPCM1.5 and PPM, compared to bulk water. **f** The recyclability of PPCM1.5-wood during 20-h solar vapor evaporation.

As a proof-of-concept, the utility of PPCM1.5 was evaluated by solar thermal evaporation (Supplementary Fig. 16). The PPCM1.5 was deposited on the surface of a natural wood to construct a bilayer solar evaporator (named as PPCM1.5-wood). The PPCM1.5-wood displays a high light absorption (90–98%) in the UV-vis-NIR regions (Fig. 6a), compared to PPM-wood (30–38%) or wood (10–15%). The high light absorption is likely due to the fine dispersion of CNT and the light multiply reflection on the hierarchical pores^48–50^. Indeed, it is found that the light absorption of a control membrane, i.e., a dense PPM contains similar amount of CNT, shows lower light absorption temperature under 1-sun irradiation (Supplementary Fig. 17). Under 1-sun irradiation, the surface temperature of PPCM1.5-wood (wet) gradually increases to ca. 53 °C (Fig. 6b), higher than that of PPM1.5-wood (ca. 45 °C), wood (ca. 42 °C) or bulk water (ca. 42 °C). Infrared imaging results (Supplementary Fig. 18) confirm the efficient solar-to-thermal capability of PPCM1.5-wood, which is beneficial for solar evaporation. The mass of water decreases linearly upon irradiation time, and the evaporation rate of PPCM1.5-wood under 1-sun irradiation is 2.8 kg/m^2^h (Fig. 6c), significantly higher than PPM-wood (1.3 kg/m^2^h), wood (1.1 kg/m^2^h), bulk water (0.8 kg/m^2^h), and a dense PPCM without pores (Supplementary Fig. 17). The evaporation rate of PPCM1.5-wood ranks one of the top-tier values among solar evaporators (Fig. 6d, Supplementary Table 1).

The high evaporation rate is related to the reduced evaporation enthalpy of PPCM1.5-wood (Supplementary Figs. 19, 20). The water evaporation enthalpy in PPCM1.5-wood is measured as 0.94 kJ/g by differential scanning calorimeter (DSC), which is reduced by 61% compared to that of bulk water (2.4 kJ/g) (Fig. 6e, Supplementary Table 2). Previous work reported that there are three types of water molecules in hydrophilic evaporators, namely, free water (FW), intermediate water (IW), and bound water^51,52^. The IW forms hydrogen bond with the bound water, which weakens the hydrogen bonds among IW molecules and reduces the energy demand for water evaporation. Raman characterization (Supplementary Fig. 21) shows that the IW:FW ratio of PPCM1.5-wood (0.53) is higher than that of pure water (0.33), proving that the existence of more IW in the PPCM1.5-wood reduce water evaporation enthalpy. Noteworthy, PPCM1.5-wood maintains good performance versus time (Fig. 6f) and salt concentration (Supplementary Fig. 22) due to the osmotic pumping of polyelectrolytes^53^. Collectively, the hydrophilic nature of polyelectrolytes is beneficial for the reduced enthalpy of water evaporation, while the hierarchical pores promotes water transport. Thus the SWOW mechanism is versatile for structure and functionality engineering of water-soluble polymers.

## Discussion

Contrast to the classic oil-on-water spreading, the water-on-water spreading has been less exploited for materials engineering and chemical synthesis. With inspiration from the skin formation mechanism, a mechanism was proposed to enable the SWOW of regular-size water droplet, without the need for specialized equipment. The PEI-PSSNa solution features smaller surface tension (*γ*_s_ = 60 mN/m) than water, which provides the driving force (Δ*γ*) for surface spreading. When the solution droplet was placed on acidic water, the PEI-PSSNa complexation was initiated because of the protonation of amine groups in acidic water. Thus, the Δ*γ* represents the driving force for SWOW, while the PEI-PSSNa interfacial complexation circumvents water-in-water mixing. The synergy of Δ*γ* and the pH-responsive polyelectrolyte complexation renders efficient SWOW, which could be controlled by tuning the Δ*γ* and complexation (*K*_c_), respectively (Fig. 4).

The SWOW mechanism is facile and generic, viable with regular-size droplets and water-soluble polymers. As a proof of concept, hybrid membranes incorporated with CNTs were prepared at ambient conditions, which feature hierarchical pores facilitating efficient solar absorption and energy conversion, e.g., the solar thermal evaporation rate of PPCM1.5 is 2.8 kg/m^2^h evaporation rate under 1-sun irradiation. Thus the SWOW mechanism enables functionality engineering of water-soluble polymers at dynamic interfaces between miscible solvents, with more opportunities remain open for future research. For example, it remains unclear how to prepare membranes with arbitrary shapes using the SWOW mechanism. In addition, the surface spreading of polyelectrolyte solution is a self-limiting process, i.e., polyelectrolytes were solidified quickly, due to which the continuous withdrawal of SWOW membranes is challenging.

## Methods

### Materials

Polyethyleneimine (PEI, *M*_w_ = 70,000, 50 wt% in H_2_O) was purchased from Aladdin Industrial Co. Ltd (China). Poly(sodium 4-styrenesulfonate) (PSSNa, *M*_w_ = 1,000,000 g mol^−1^) was purchased from Sigma-Aldrich Industrial Co. Ltd (China). CNTs were purchased from Shenzhen Nanoport Co. Ltd (China). Sodium hydroxide, hydrochloric acid (HCl), tetrahydrofuran, ethanol, isopropanol, and acetone were purchased from Sinopharm Chemical Reagent Co. Ltd (China).

### Preparation of PEI-PSSNa membranes

1.54 g of PSSNa and 1.93 g PEI (50 wt% in H_2_O) were dissolved in 6.5 mL H_2_O, stirred to form a homogeneous PEI-PSSNa solution. A drop of the PEI-PSSNa solution was placed on surface of water (note: pH of water was adjusted by using 6 M HCl). The droplet quickly spread on the water surface to form a free-floating PEI-PSSNa membrane, which was named as PPMx (x denotes the pH of water). Taking PPM1.5 for example, the mass retention of PEI and PSS polymers is ~75%. The preparation of PEI-PSSNa-CNT membranes (PPCMx) follows the same procedure, except that CNTs were dispersed in PEI-PSSNa solution.

### Solar thermal evaporation experiments

A solar light simulator (CEL-S500L) was used to conduct the solar thermal steaming measurement at ca. 40 °C room temperature and 30% relative humidity. The surface temperature of samples was monitored by the infrared camera. The water mass changes were measured by an electronic balance (JA2003, Soptop). The evaporation rate (kg/m^2^h) was calculated by the following equation: evaporation rate = Δ*m* ⁄ (S*t), where Δ*m* is the mass change of water in 1 h under 1-sun irradiation, *S* is the area (m^2^), and t represents the time of solar irradiation.

### Characterizations

The morphologies of PPMs were observed by electron microscopy (FESEM, SU-8010), atomic force microscopy (AFM, SPM-9700), Ultra-Depth 3D Microscope (VHX-1000C) and Cryogenic scanning electron microscopy (Cryo-SEM, FEI Quanta 450). Fourier transform infrared spectroscopy (FT-IR) were obtained by a BRUKER Vertex 80 FT-IR (Germany). The interfacial tension of droplets and contact angle of films were measured by a contact angle meter (DATAPPHYSICS, OCA20). Element analyses (C, N, S, and H) were analyzed by an element analyzer (PerkinElmer, Optima 5300-DV). UV-Vis-NIR was conducted on a spectrophotometer (Lambda 750 S). Raman microscopy (LabRam HR Evolution) was recorded at 532 nm. X-ray photon spectroscopy (XPS) was recorded on ESCALAB Xi^+^ instrument (Thermo Fischer, Al Ka irradiation, 12.5 kV working voltage). For depth-dependent XPS measurements, PPM2.25 was etched using argon-ions (3000 eV) for different time. Differential scanning calorimeter (DSC) was done with DSC2500. Inductively coupled plasma emission (ICP-OES) was conducted with a PerkinElmer 8300 spectrometer. pH values were measured by a digital pH meter (SARTORIUS, pB-10).

## Supplementary information

Supplementary information

Peer Review File

Description of Additional Supplementary Files

Supplementary Movie 1

## Data Availability

Data available on request from the corresponding author.
