# Supplementary material for: Spontaneous water-on-water spreading of polyelectrolyte membranes inspired by skin formation
Source: Nat Commun. 2022 Jun 9;13:3227. doi: 10.1038/s41467-022-30973-6 (PMC9184545; doi:10.1038/s41467-022-30973-6)
Supplement: Supplementary file 1 — Supplementary information [file 41467_2022_30973_MOESM1_ESM.pdf]

Supplementary Information

Spontaneous water-on-water spreading of polyelectrolyte membranes inspired by skin formation

Sihan Tang, Jiang Gong, Yunsong Shi, Shifeng Wen and Qiang Zhao*

Key Laboratory of Material Chemistry for Energy Conversion and Storage, Ministry of Education, School of Chemistry and Chemical Engineering, Huazhong University of Science and Technology Luoyu Road No. 1037, 430074, Wuhan (China).

State Key Laboratory of Materials Processing and Die & Mould Technology, Huazhong University of Science and Technology Luoyu Road No. 1037, 430074, Wuhan (China).

Department of Orthopaedics, Union Hospital, Tongji Medical College, Huazhong University of Science and Technology, 430022, Wuhan (China).

1. Preparation of PP aqueous solution and characterizations of PPMs

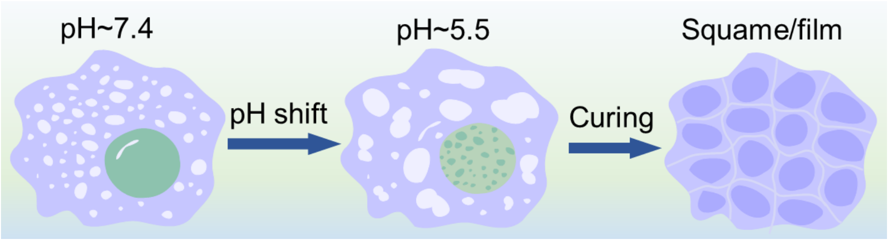

Supplementary Fig. 1. Schematic process of the pH-responsive water-water interfaces in the formation of skin barriers. Please note: a sudden shift in intracellular pH occurs near the skin surface, which regulates liquid-phase dynamics to drive squame formation (Science, 2020, 367, 1210).

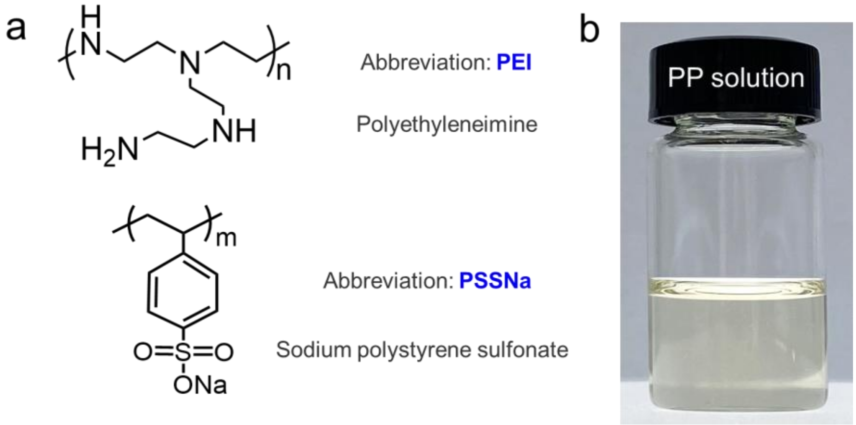

Supplementary Fig. 2. (a) Chemical structures of polyethyleneimine (PEI) and sodium polystyrene sulfonate (PSSNa), (b) An optical picture of a PEI-PSSNa (abbreviation: PP) solution (25 wt%).

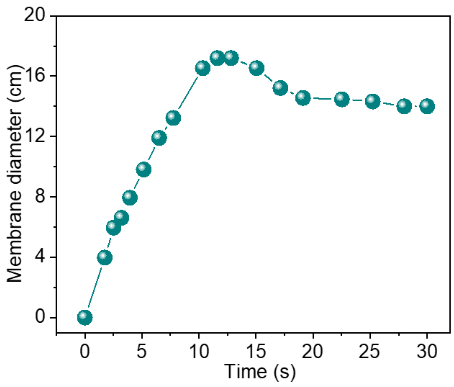

Supplementary Fig. 3. Effect of spreading time on diameter of PPM2.25. Please note: the volume of PEI-PSSNa droplet is ~140 μL.

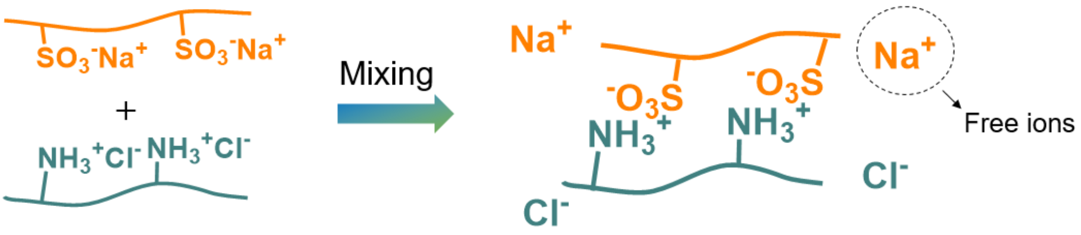

Supplementary Fig. 4. Schematic illustration of the PEI-PSSNa complexation and the release of Na^+ counter ions. Please note: the content of Na^+ in PPMs is very low (~ 0.1 wt%)

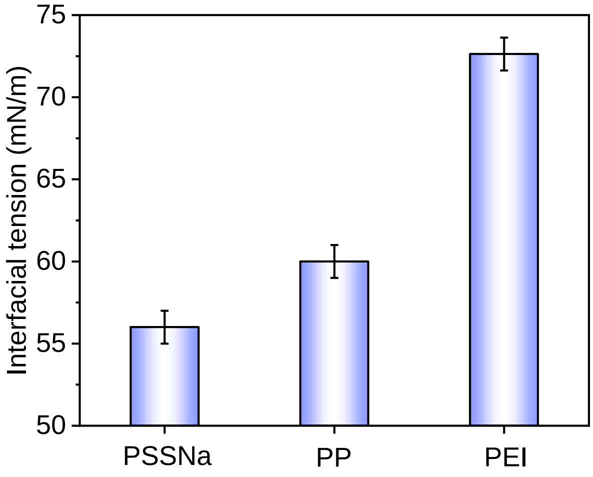

Supplementary Fig. 5. Interfacial tension of 25 wt% PEI, PSSNa and PP solution respectively. Please note: PP represents PEI-PSSNa solution. Error bars: standard deviation.

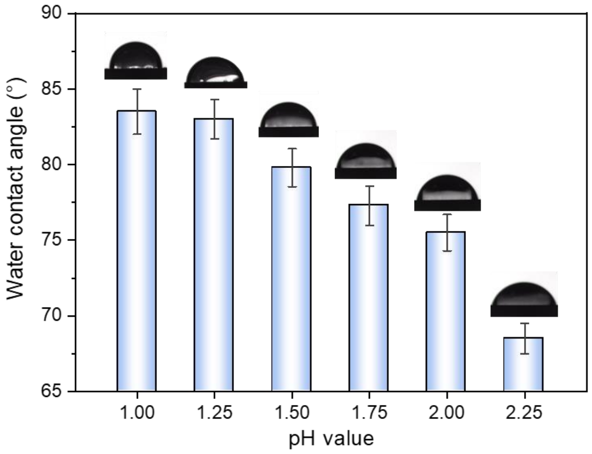

Supplementary Fig. 6. Water contact angle of PPM*x* (pH 1-2.25).

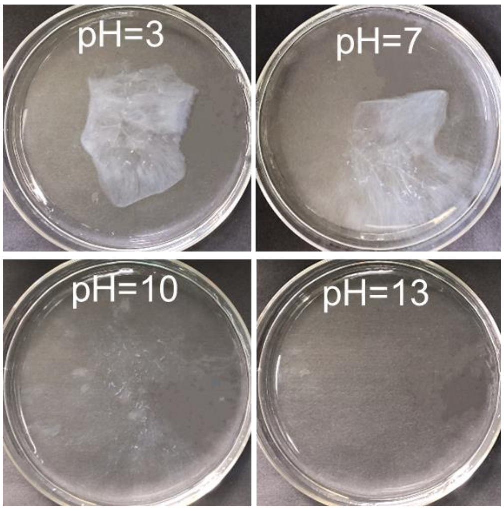

Supplementary Fig. 7. Optical pictures of 25 wt% PP solution spread on water with different pH (3, 7, 10 and 13). Please note: at pH 3, a fluidic film form on top of water surface, because the PEI-PSSNa complexation is not strong enough to enable the solidification of PEI-PSSNa membrane. Diameter of the glass container is 16 cm.

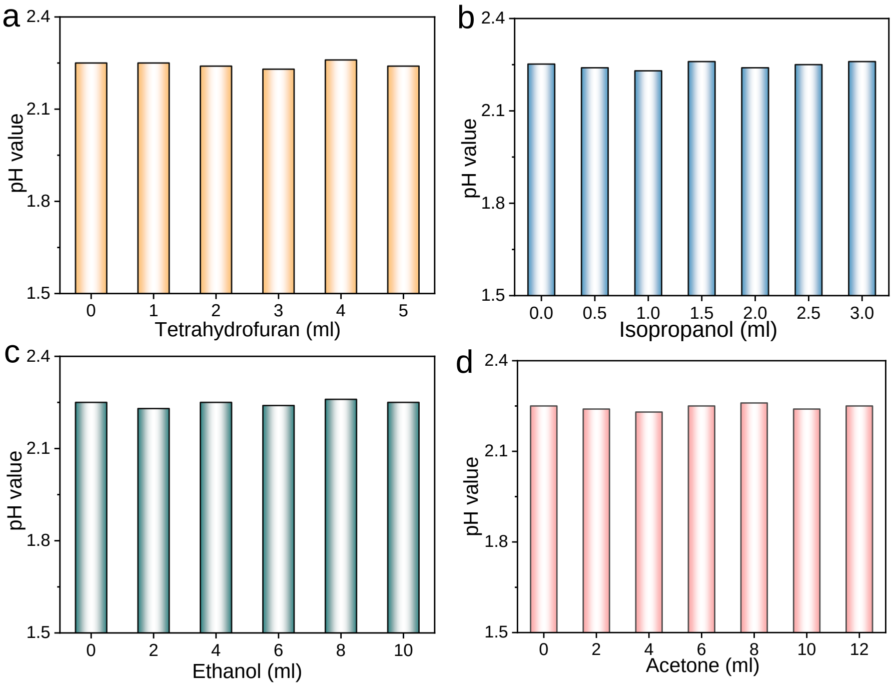

Supplementary Fig. 8. Effect of adding amount of (a) Tetrahydrofuran, (b) Isopropanol, (c) Ethanol, and (d) Acetone in the pH value organic-water mixture solvent. Note: the initial pH value and volume of water (without adding solvents) is maintained at 2.25 and 250 mL.

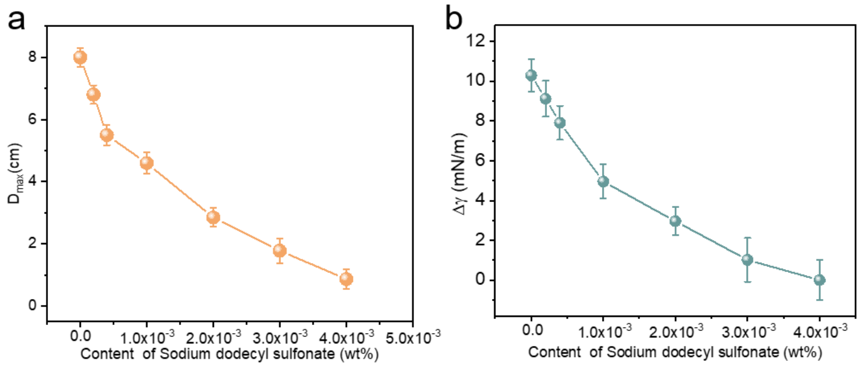

Supplementary Fig. 9. Effect of sodium 1-dodecanesulfonate content in (a) D_{max} and (b) $\Delta\gamma$ (γ_w-γ_s) of PPM1.75. Error bars: standard deviation.

2. Generic utility of the SWOW spreading mechanism

2.1. SWOW spreading of the PDDA-PSSNa system triggered by salt ions.

Preparation of PDDA-PSSNa solution (0.75 M). 1.55 g of PSSNa (M_w = 70,000 g mol^{-1}) and 2.93 g NaCl were dissolved in 10 mL H_2O. 6.06 g PDDA (20 wt% in H_2O, M_w= 400,000-500,000 g, Sigma Aldrich) and 2.93 g NaCl were dissolved in 5 mL H_2O. The two solutions were mixed under vigorous stirring (600 rpm) to form a homogenous solution. A drop of the PDDA-PSSNa solution was placed on DI water. The solution spread on the water to form a PDDA-PSSNa membrane (Supplementary Fig. 10).

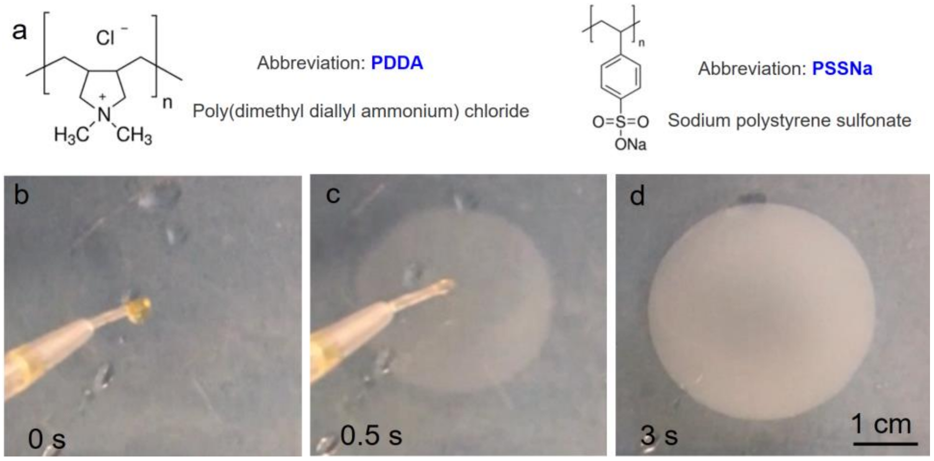

Supplementary Fig. 10. (a) Chemical structures of PDDA and PSSNa polyelectrolytes, (b-d) optical picture of a drop of PDDA-PSSNa aqueous solution placed on surface of DI water. Please note: insertion is spreading time: 0 s, 0.5 s and 3 s.

Discussions. The PDDA-PSSNa complexation was screened by concentrated NaCl (5 M). When the solution was placed on DI water, the Na^+ and Cl^- in PDDA-PSSNa

solution will quickly diffuse into DI water, thus the NaCl concentration in PDDA-PSSNa solution was decreased. Consequently, screening effect of NaCl was reduced, resulting in the PDDA-PSSNa complexation. Thus the mechanism of water-on-water spreading of PDDA-PSSNa system trigger by salt is similar to that of SWOW discussed in the main text.

2.2. *SWOW spreading of the PCMVIm-PAA system triggered by solvent exchange.*

Preparation of *PCMVIm-PAA* solution. The PCMImTf$_2$N polymer was synthetized according our previous method[1]. Molecular weight of the polymer is 20 000, and the molecular weight distribution is 2.1. Polyacrylic acid (PAA, Mw: 2000 g mol^{-1}) was purchased from Sigma Aldrich. PCMImTf$_2$N (1.0 g) and PAA (0.14 g) was dissolved in dimethylformamide (DMF) solvent to form a homogenous solution (10 wt%). A droplet of the PCMImTf$_2$N-PAA solution was placed on water with different pH.

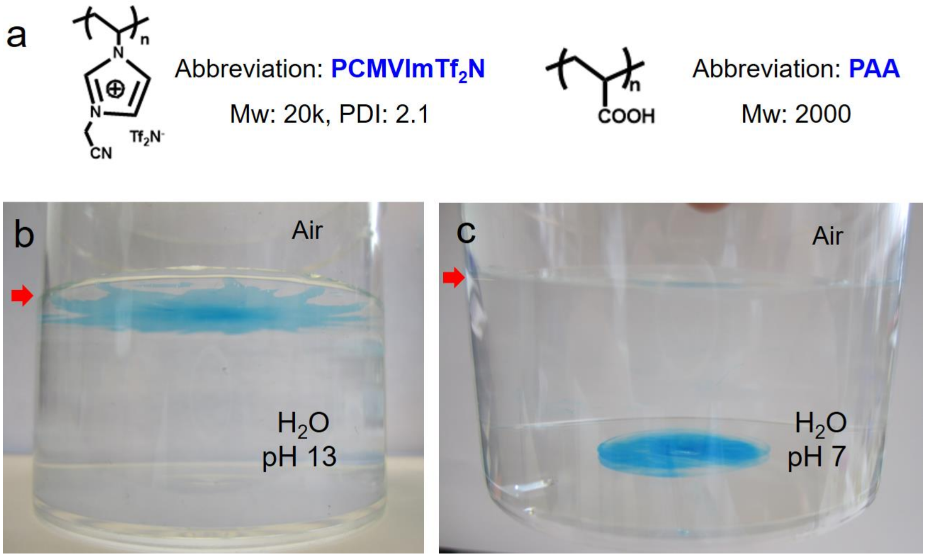

Supplementary Fig. 11 (a) Chemical structures of PCMImTf$_2$N and PAA polyelectrolytes. (b, c) Optical photographs of a drop of PCMVImTf$_2$N-PAA/DMF solution placed on top of (b) H_2O (pH 13) and (c) H_2O (pH 7). Please note: trace amount of methyl blue was added in the PCMImTf$_2$N-PAA/DMF solution to make it more visible. The red arrow indicates the position of air-water interfaces. Diameter of the container (b,c) is ca. 5 cm.

Discussions: The PCMVImTf$_2$N-PAA mixture solution spread on top of the alkali water (pH 13, Supplementary Fig. 11b), while it sinks to the bottom of water (pH 7, Supplementary Fig. 11c). The acrylic acid groups (COOH) was NOT deprotonated in DMF. When the solution was placed on alkali water (Supplementary Fig. 11b), the PCMVImTf$_2$N-PAA complexation was initiated due to the higher pH of water which deprotonate COOH groups, *i.e.,* from COOH groups to COO- groups. Then the concurrent PCMVImTf$_2$N-PAA complexation stabilizes water-water interface and facilitate SWOW spreading. By contrast, the PCMVImTf$_2$N-PAA complexation was NOT effectively initiated by neutral water (Supplementary Fig. 11c), and the solution sinks into water, without spreading. Thus the spreading of PCMVImTf$_2$N-PAA system follow similar principle of SWOW in the main text.

3. The preparation and solar-thermal evaporation of PPCM

Preparation of PPC solution: 3.04 g of PSSNa, 3.82 g PEI (50 wt% in H_2O) and 0.15 g CNT were added in 18.1 mL H_2O, and the solution was mixed by ultrasonic processor for 2 h. The solution droplet was placed on acidic water to prepare the PPCMs. The PPCM1.5 (6 pieces) were chosen, and transferred onto porous logs (~3 cm in diameter) for solar thermal evaporation experiments.

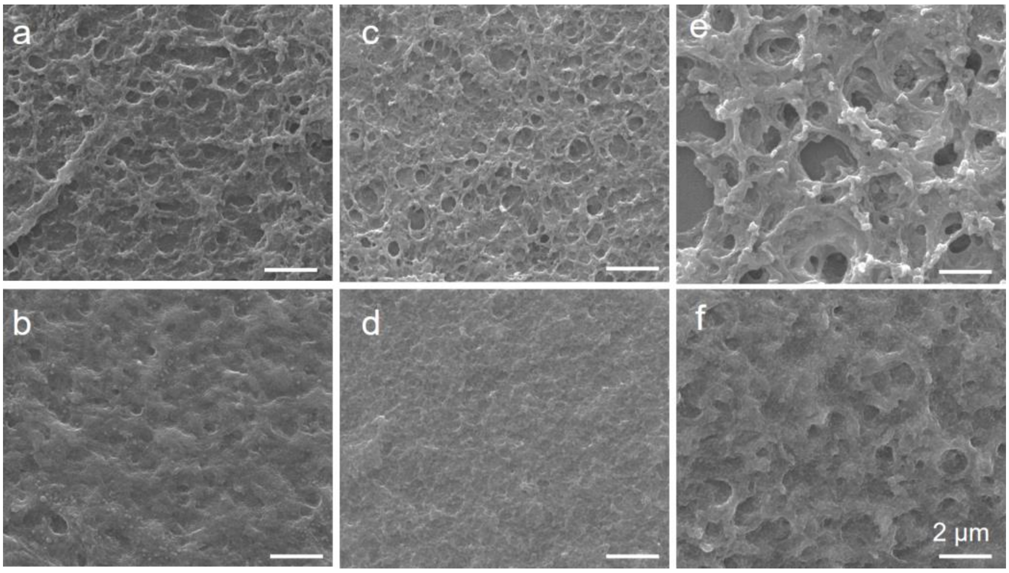

Supplementary Fig. 12. Cryo-SEM images of (a,b) PPCM2.25, (c,d) PPCM2, (e,f) PPCM1.75. All scale bars are 2 μm. Images at (a,c,e) and (b,d,f) represent top (contacting with air) and bottom (contacting with water) surfaces of PPCMs.

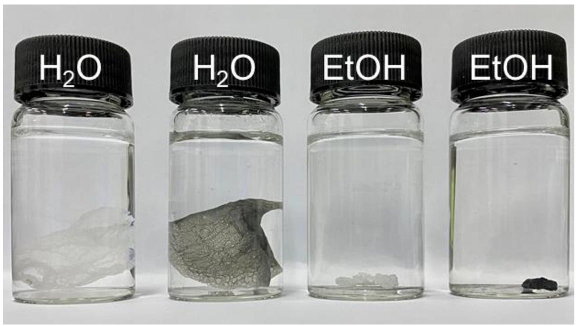

Supplementary Fig. 13. Optical images of PPM and PPCM1.5 (black) in water or ethanol.

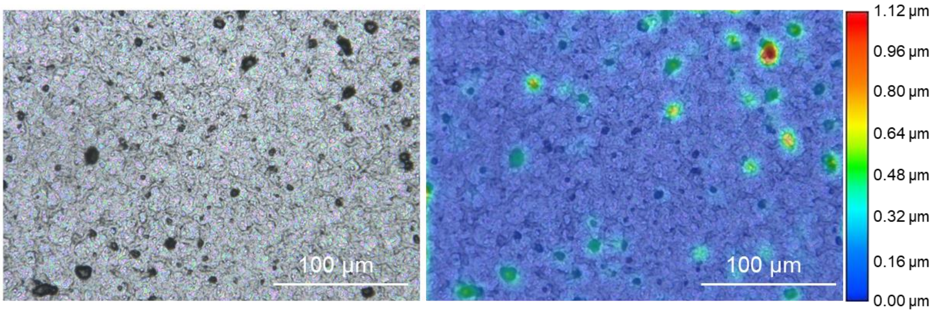

Supplementary Fig. 14. Surface morphology of PPCM measured by ultra-depth 3D Microscope.

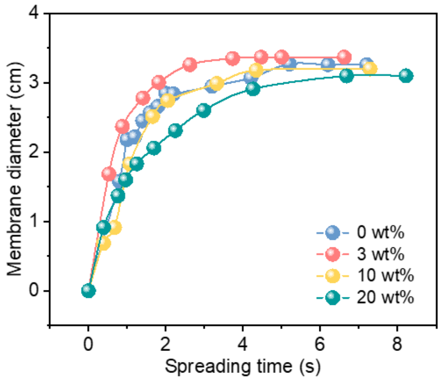

Supplementary Fig. 15. Dependence of membrane diameter on the CNT content in the PEI-PSSNa droplet. Please note: the droplet was spread on pH 1.5 water.

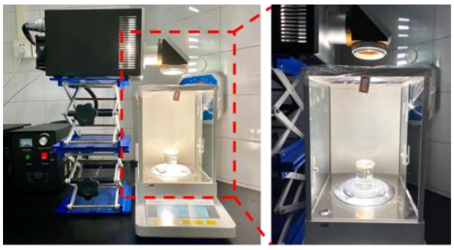

Supplementary Fig. 16. Digital images of interfacial solar-driven steam generation instrument.

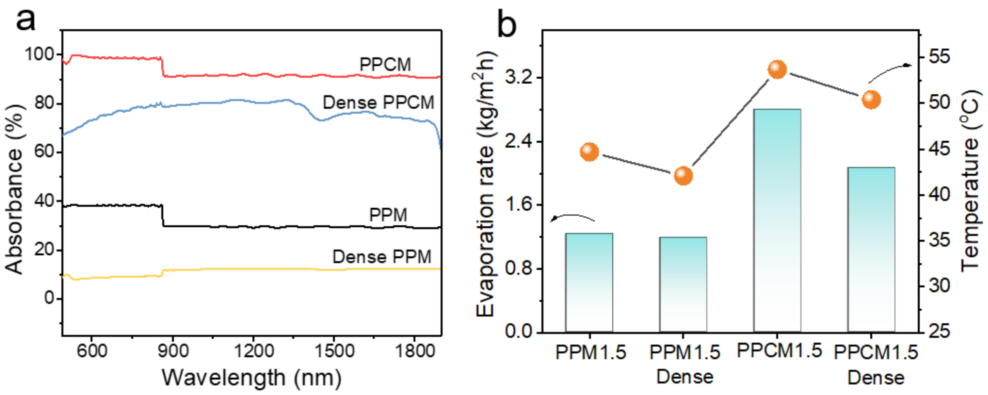

Supplementary Fig. 17. (a) UV-vis absorption and (b) water evaporation rate (1-sun irradiation) of PPM1.5, dense PPM1.5, PPCM1.5, and dense PPCM1.5. Please note: dense PPMs were prepared by casting the PEI-PSSNa (or PEI-PSSNa-CNT) solution on glass plates, following by drying (50 °C, 2h) and acid annealing.

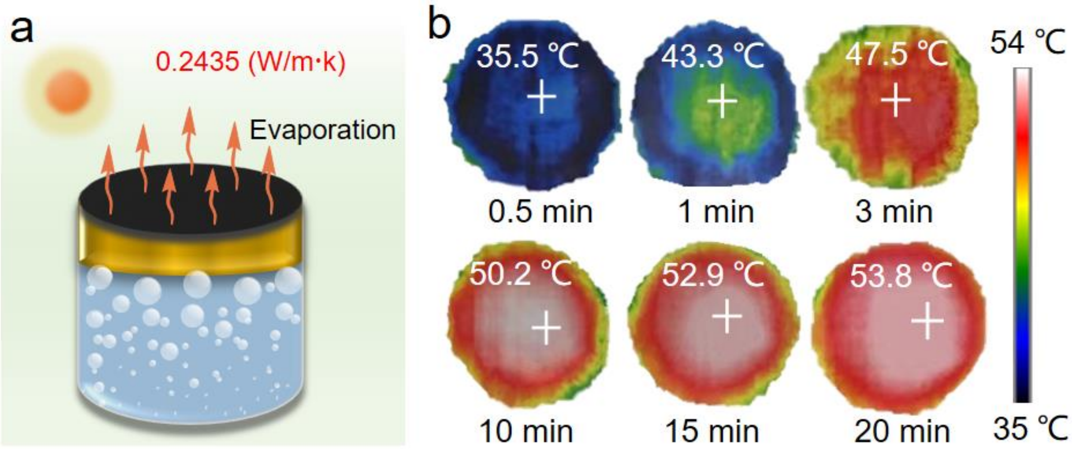

Supplementary Fig. 18. (a) Schematic diagram of solar-driven steam evaporation. (b) Infrared images of PPCM1.5 under 1-sun irradiation.

Supplementary Table 1 Comparison of evaporation rate of PPCM1.5 with literature.

Entry	Evaporation rate (kg·m^{-2}·h^{-1})	Efficiency (%)	Reference in supporting information
**1**	**2.8**	**70**	**This work**
2	2.06	90.5	[2]
3	2	85.7	[3]
4	1.83	82.2	[4]
5	1.68	97	[5]
6	1.38	90.8	[6]
7	1.34	89.4	[7]
8	1.12	82.2	[8]
9	1.3	89	[9]
10	1.38	86.9	[10]
11	1.69	94.9	[11]
12	2.62	108.1	[12]
13	2.2	87	[13]
14	2.08	97	[14]
15	1.85	90	[15]
16	1.8	90	[16]
17	1.55	92.6	[17]
18	1.69	97.1	[18]
19	1.68	80	[19]
20	2.15	76.2	[20]
21	1.8	95.5	[21]
22	1	68	[22]
23	1.2	84	[23]
24	1.36	86	[24]
25	2.38	99.9	[25]
26	1.2	84	[26]
27	2.8	92	[27]

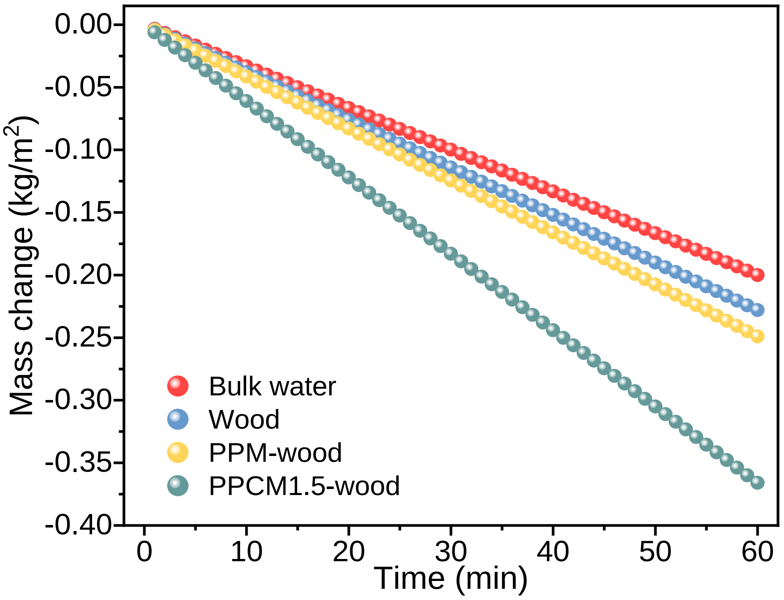

Supplementary Fig. 19. Mass changes of water over of PPCM1.5, wood, PPM-wood, PPCM1.5-wood in dark.

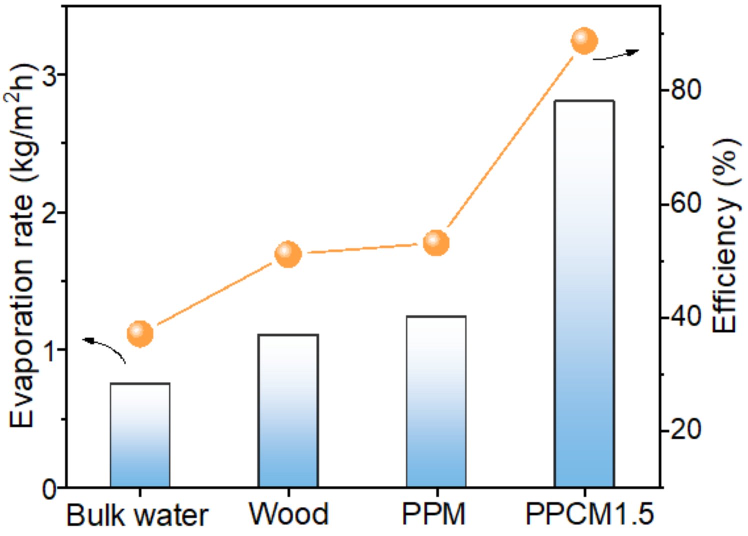

Supplementary Fig. 20. Evaporation rates and conversion efficiency of bulk water, wood, PPM and PPCM1.5.

Supplementary Table 2 Summary of the enthalpy values measured from DSC measurement and dark experiment.

Enthalpy (J g^{-1})	Pure water	PPM	PPCM1.5
DSC measurement	2400	1970	940
Dark experiment	2402	1929	1313

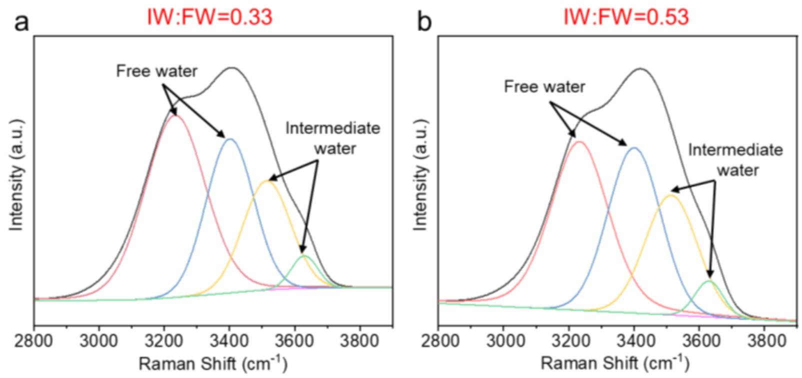

Supplementary Fig. 21. Raman spectra of (a) the bulk water and (b) PPCM1.5 with the fitted peaks showing IW and FW.

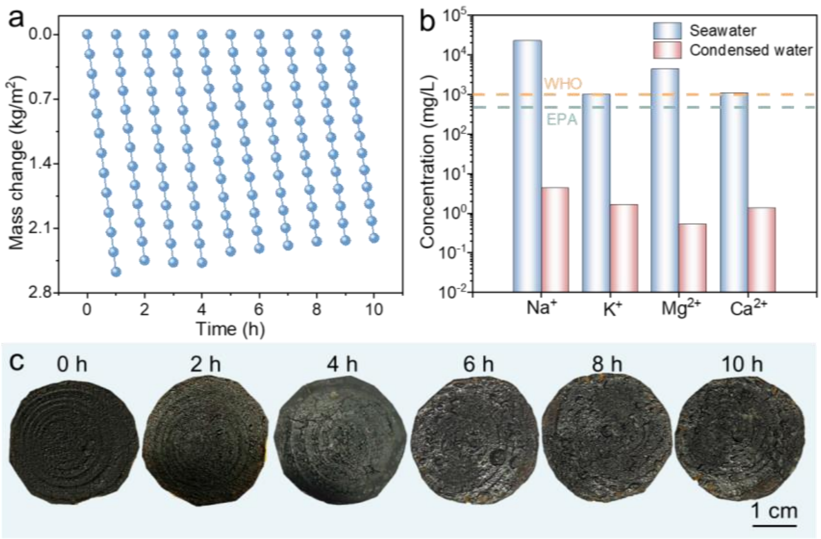

Supplementary Fig. 22. (a) Solar-driven steam evaporation of seawater under 1 Sun irradiation. (b) Concentration of ions in pristine seawater and the condensed water collected during the solar evaporation of PPCM1.5 monolith. (c) Optical picture of salt precipitation on the evaporator surface after 10 h cycles. The seawater for solar thermal tests was collected from the Bohai Sea (China).

REFERENCES

1 Zhao, Q., Zhang, P., Antonietti, M. & Yuan, J. Poly(ionic liquid) complex with spontaneous micro-/mesoporosity: template-free synthesis and application as catalyst support. *J Am Chem Soc* **134**, 11852-11855, doi:10.1021/ja303552p (2012).

2 Hu, G. *et al.* Salt-Resistant Carbon Nanotubes/Polyvinyl Alcohol Hybrid Gels with Tunable Water Transport for High-Efficiency and Long-Term Solar Steam Generation. *Energy Technology*, 1900721, doi:10.1002/ente.201900721 (2019).

3 Xu, W. *et al.* Efficient Water Transport and Solar Steam Generation via Radially, Hierarchically Structured Aerogels. *ACS nano* **13**, 7930-7938, doi:10.1021/acsnano.9b02331 (2019).

4 Tan, M., Wang, J., Song, W., Fang, J. & Zhang, X. Self-floating hybrid hydrogels assembled with conducting polymer hollow spheres and silica aerogel microparticles for solar steam generation. *Journal of Materials Chemistry A* **7**, 1244-1251, doi:10.1039/c8ta10057h (2019).

5 Zhang, B. *et al.* Molten salts promoting the "controlled carbonization" of waste polyesters into hierarchically porous carbon for high-performance solar steam evaporation. *Journal of Materials Chemistry A* **7**, 22912-22923, doi:10.1039/c9ta07663h (2019).

6 Ma, X. *et al.* Hierarchical Porous SWCNT Stringed Carbon Polyhedrons and PSS Threaded MOF Bilayer Membrane for Efficient Solar Vapor Generation. *Small*, e1900354, doi:10.1002/smll.201900354 (2019).

7 Qin, D.-D., Zhu, Y.-J., Chen, F.-F., Yang, R.-L. & Xiong, Z.-C. Self-floating aerogel composed of carbon nanotubes and ultralong hydroxyapatite nanowires for highly efficient solar energy-assisted water purification. *Carbon* **150**, 233-243, doi:10.1016/j.carbon.2019.05.010 (2019).

8 Liu, S., Huang, C., Huang, Q., Wang, F. & Guo, C. A new carbon-black/cellulose-sponge system with water supplied by injection for enhancing solar vapor generation. *Journal of Materials Chemistry A* **7**, 17954-17965, doi:10.1039/c9ta02913c (2019).

9 Chen, Z. *et al.* Deep Eutectic Solvent-Assisted In Situ Wood Delignification: A Promising Strategy To Enhance the Efficiency of Wood-Based Solar Steam Generation Devices. *ACS Appl Mater Interfaces* **11**, 26032-26037, doi:10.1021/acsami.9b08244 (2019).

10 Chen, J. *et al.* Janus Evaporators with Self-Recovering Hydrophobicity for Salt-Rejecting Interfacial Solar Desalination. *ACS Nano*, doi:10.1021/acsnano.0c07677 (2020).

11 Shao, B. *et al.* A general method for selectively coating photothermal materials on 3D porous substrate surfaces towards cost-effective and highly efficient solar steam generation. *Journal of Materials Chemistry A* **8**, 24703-24709, doi:10.1039/d0ta08539a (2020).

12 Xu, Y. *et al.* Low cost, facile, environmentally friendly all biomass-based squid

ink-starch hydrogel for efficient solar-steam generation. *Journal of Materials Chemistry A* **8**, 24108-24116, doi:10.1039/d0ta08620g (2020).
13 Zhang, X. *et al.* Nature-inspired design: p- toluenesulfonic acid-assisted hydrothermally engineered wood for solar steam generation. *Nano Energy* **78**, doi:10.1016/j.nanoen.2020.105322 (2020).
14 Chen, S. *et al.* Plasmonic wooden flower for highly efficient solar vapor generation. *Nano Energy* **76**, doi:10.1016/j.nanoen.2020.104998 (2020).
15 Tang, J. *et al.* Realization of Low Latent Heat of a Solar Evaporator via Regulating the Water State in Wood Channels. *ACS Appl Mater Interfaces* **12**, 18504-18511, doi:10.1021/acsami.0c01261 (2020).
16 Wang, Z. *et al.* Versatile coating with multifunctional performance for solar steam generation. *Nano Energy* **74**, doi:10.1016/j.nanoen.2020.104886 (2020).
17 Xu, Y. *et al.* A simple and universal strategy to deposit Ag/polypyrrole on various substrates for enhanced interfacial solar evaporation and antibacterial activity. *Chemical Engineering Journal* **384**, doi:10.1016/j.cej.2019.123379 (2020).
18 Zheng, Z. *et al.* High-absorption solar steam device comprising Au@Bi2MoO6-CDs: Extraordinary desalination and electricity generation. *Nano Energy* **68**, doi:10.1016/j.nanoen.2019.104298 (2020).
19 Irshad, M. S. *et al.* Salt-resistant carbon dots modified solar steam system enhanced by chemical advection. *Carbon* **176**, 313-326, doi:10.1016/j.carbon.2021.01.140 (2021).
20 Shi, Y., Ilic, O., Atwater, H. A. & Greer, J. R. All-day fresh water harvesting by microstructured hydrogel membranes. *Nat Commun* **12**, 2797, doi:10.1038/s41467-021-23174-0 (2021).
21 Wu, F. *et al.* Bayberry tannin directed assembly of a bifunctional graphene aerogel for simultaneous solar steam generation and marine uranium extraction. *Nanoscale* **13**, 5419-5428, doi:10.1039/d0nr08956g (2021).
22 Wu, X. *et al.* A thermally engineered polydopamine and bacterial nanocellulose bilayer membrane for photothermal membrane distillation with bactericidal capability. *Nano Energy* **79**, doi:10.1016/j.nanoen.2020.105353 (2021).
23 Huang, C. H. *et al.* Tailoring of a Piezo-Photo-Thermal Solar Evaporator for Simultaneous Steam and Power Generation. *Advanced Functional Materials*, doi:10.1002/adfm.202010422 (2021).
24 Zou, Y. *et al.* A Mussel-Inspired Polydopamine-Filled Cellulose Aerogel for Solar-Enabled Water Remediation. *ACS Appl Mater Interfaces* **13**, 7617-7624, doi:10.1021/acsami.0c22584 (2021).
25 Xia, Y. *et al.* Rational designs of interfacial-heating solar-thermal desalination devices: recent progress and remaining challenges. *Journal of Materials Chemistry A* **9**, 6612-6633, doi:10.1039/d0ta11911c (2021).
26 Huang, C. H. *et al.* Tailoring of a Piezo-Photo-Thermal Solar Evaporator for Simultaneous Steam and Power Generation. *Advanced Functional Materials* **31**, doi:10.1002/adfm.202010422 (2021).
27 Zhu, L. *et al.* A solution to break the salt barrier for high-rate sustainable solar

desalination. *Energy & Environmental Science* **14**, 2451-2459, doi:10.1039/d1ee00113b (2021).
