# Supplementary material for: Spontaneous water-on-water spreading of polyelectrolyte membranes inspired by skin formation
Source: Nat Commun. 2022 Jun 9;13:3227. doi: 10.1038/s41467-022-30973-6 (PMC9184545; doi:10.1038/s41467-022-30973-6)
Supplement: Supplementary file 3 — Description of Additional Supplementary Files [file 41467_2022_30973_MOESM3_ESM.pdf]

Description of Additional Supplementary Files

File Name: Supplementary Movie 1
Description: Video of a PEI-PSSNa droplet spreading on pH 2.25 water
