# Supplementary material for: Spontaneous water-on-water spreading of polyelectrolyte membranes inspired by skin formation
Source: Nat Commun. 2022 Jun 9;13:3227. doi: 10.1038/s41467-022-30973-6 (PMC9184545; doi:10.1038/s41467-022-30973-6)
Supplement: Supplementary file 2 — Peer Review File [file 41467_2022_30973_MOESM2_ESM.pdf]

nature portfolio

Peer Review File

Spontaneous water-on-water spreading of polyelectrolyte membranes inspired by skin formation

Open Access This file is licensed under a Creative Commons Attribution 4.0 International License, which permits use, sharing, adaptation, distribution and reproduction in any medium or format, as long as you give appropriate credit to the original author(s) and the source, provide a link to the Creative Commons license, and indicate if changes were made. In the cases where the authors are anonymous, such as is the case for the reports of anonymous peer reviewers, author attribution should be to 'Anonymous Referee' followed by a clear attribution to the source work. The images or other third party material in this file are included in the article's Creative Commons license, unless indicated otherwise in a credit line to the material. If material is not included in the article's Creative Commons license and your intended use is not permitted by statutory regulation or exceeds the permitted use, you will need to obtain permission directly from the copyright holder. To view a copy of this license, visit http://creativecommons.org/licenses/by/4.0/.

Reviewers' Comments:

Reviewer #1:
Remarks to the Author:
In the manuscript entitled “Spontaneous, water-on-water spreading and assembly of polyelectrolytes inspired by skin formation”, the authors proposed a water on water spreading method to construct porous polyelectrolyte films based on the complexation induced solidification mechanism. The spreading process, the influence of surface tension difference and pH on the spreading diameter, and the versatility of this method salt-triggered and solvent exchange-triggered film formation system were investigated. Finally, the authors also showed the application of this method to prepare CNT loaded porous film for solar evaporation. This work is interesting with a relatively clear and systematic logic. However, I still have some reservations, on addressing which the article can be improved further.

1. The characterization of the porous film.
Only surface morphology of the prepared films is provided without the indication whether the characterized surface is the bottom interface contacting with water or the top surface contacting with air. Both surfaces should be characterized. The cross-sectional characterization of the porous film is suggested. In addition, the characterization in the thickness direction should also be provided to indicate the film uniformity.
2. The formation mechanism focuses only on the diameter. The thickness or thickness profile change, i.e., the complexation mechanism in the thickness direction, with the variation of pH or should also be investigated. Will the membrane gradually contracting process influence the thickness uniformity?
3. Coffee ring-like structure occurs in the CNT loaded film which leads to an uneven film. Does this phenomenon occur only in the CNT loaded the system or also in the CNT absent system? Is this related with the membrane gradually contracts process?

Reviewer #2:
Remarks to the Author:
The authors prepared polyelectrolyte complex membranes in acidic water using PSS and PEI and studied the solar thermal evaporation performance. The resultant polyelectrolyte complex membranes are well characterized. The methodology is sound and can be reproduced. The article has some typos which need to be corrected.

The authors have claimed that they are the first to engineer such porous membranes, which is not true. There is a report in the literature that has studied the exact same polyelectrolytes for porous membranes production and has fine-tuned their properties. The reference to their work is missing (J. Mem. Sci. 2021, 625, 11911).

Furthermore, the authors have only ‘mentioned’ the results without providing sufficient reasoning. For example, why does the film diameter increase in the beginning and then decrease? Why does the PPM2.25 membrane have smaller pores as compared to PPCM1.5 membranes even though the latter was prepared at lower pH values? How does the addition of CNT affect the membrane formation kinetics and why does it retain the higher pore size? How does CNT attach to the polyelectrolyte complex? The discussion section severely lacks such reasoning and is only a summary at the moment.

Therefore, in light of these comments, I cannot recommend the publication of this article in Nature communications in its current form.

Specific Comments:
1. The authors should mention in the introduction why PSS and PEI were specifically chosen for this application?

2. Porous PSS-PEI membranes are already reported in the literature. The authors have followed a similar protocol for the preparation of polymer solutions and subsequent precipitation. Please add the relevant reference.

3. Line 118. The word 'mini-seconds' is not scientific and should be removed. The ref 36 which it corresponds to studies sodium polyacrylate (SPA) and polyallylamine hydrochloride (PAH) complexation as opposed to PSS-PEI studied in this work. The kinetics are very different for these complexes and are a function of several variables including the pH of the solution and water bath. Reference on PSS-PEI complexation and its kinetics would be more accurate.

4. The statement on page 6 line 121 is misleading. The polyelectrolyte complexation on liquid-liquid interfaces has been studied by several groups and there are numerous articles on this topic. (ACS Appl. Mater. Interfaces 2019, 11, 17, 16018–16026) (ACS Appl. Polym. Mater. 2020, 2, 7, 2612–2621) (J. Mem. Sci. 2021, 625, 11911).

5. Why pH 1.5 for CNT and why 2.25 for PP? The authors said that reducing pH results in stronger complexation. Then why the pores are larger for 1.5 than 2.25? The authors correlate this to freeze-drying process which is unfair. Here, complexation is the main mechanism and the authors need to verify the effect of CNT on the PSS-PEI complexation. Could it be that the CNTs are charged and interfering in the complexation? More elaborate reasoning is required.

Reviewer #3:
Remarks to the Author:
Tang et al. present a simple and efficient method of making thin polyelectrolyte films by interfacial precipitation of spreading droplets. The formation of membranes by interfacial precipitation is well known, but the mechanism described in this paper includes in a novel way spontaneous, water-on-water spreading (SWOW) to form membranes with hierarchical pores (claimed to be thinner and more uniform compared to other membranes made by bulk precipitation). The resulting hierarchical porosity is interesting and potentially quite useful. The method is much faster, though likely less precise than the common polyelectrolyte layer-by-layer deposition. The material is overall publishable in a journal of this magnitude, but presently has a number of drawbacks that need to be addressed before a decision of publication can be made. These drawbacks include:

1. The authors present data on membrane diameter, but do not present the other critical data on membrane thickness and thickness inhomogeneity. The thickness is obviously larger at the periphery, and the membranes show significant wrinkling. The control over the thickness and uniformity of the films should be presented with data and analysis.

2. The authors use the values of the static equilibrium surface tension to describe the process of dynamic spreading, during which the interfacial tensions can vary and approach the equilibrium one towards the end. This difference and the assumptions used in data interpretation should be explained carefully.

3. The data for photothermal evaporation are interesting, but unclearly described. The major parameter for comparison between the PPCM and PPM films should be the amount of CNTs deposited per unit area, which is hard to estimate. Only then one can make a conclusion whether the special porosity contributes to the more effective light capture as claimed in the paper. Also, the authors should provide more information of how CNT affects mechanical properties of the membrane (assuming they may act as structural fillers).

4. The paper is in some places written in hard to read style, and contains unclear sentences, buzzword

phrases, trivialities, and some exaggerations. This is especially problematic for the discussion section. The whole paper should be revised carefully to match the requirements for clarity and precise expression expected for a journal of such magnitude.

5. The method suffers from some disadvantages, namely the inability to make membranes of arbitrary shapes, disruption by potential interfacial instabilities, uneven thickness at periphery, difficulty in adapting to continuous film withdrawal. These disadvantages are not critical, but should be mentioned clearly, presenting the method in a balanced manner.

Reviewer #4:
Remarks to the Author:
Liquids interface is interdisciplinary topics widely important for chemistry, materials and engineering. This work represents a concrete step beyond the conventional context of oil-water spreading, and manages water-water spreading and materials functionality ingeniously. Authors stabilized miscible interfaces by pH responsive polyelectrolyte complexation featuring quick kinetics. The entire experiments were conducted with rational procedures, and the formation mechanism of the porous membranes has also been well demonstrated. Importantly, this work provided an inspiring approach to tackle the tricky water-water interfaces that quickly mix. Authors rationally propose and verify the mechanism of the WOW spreading. They also demonstrated the implication of WOW spreading for scalable preparing porous membranes with excellent solar thermal evaporation. The strength of this work lies in the WOW mechanism underpinning wide implications in chemistry and materials. Given the broad interest and high standard of nature communications, I recommend its publication in this journal pending revisions beneath.

1. The responsive PSS-PEI complexation is the key to support WOW spreading. Authors tried to prove the complexation by FT-IR which indicates the protonation and deprotonation of amine groups. Though this data is useful, could authors provide complementary data to support electrostatic complexation in a more straightforward manner?
2. Gravity of the solution droplet also plays a role in spreading. What is the density of the PSS-PEI solution, higher or lower than water?
3. Authors proposed two sets of control experiments (Fig. 3) to support the WOW mechanism. While the mechanism is convincing in general, the reviewer is curious to know the effect of more factors in the WOW process, such as adding surfactants that could be easily imagined?
4. Authors mentioned the membrane contraction in the second stage of spreading. What is the reason for membrane contraction? Is the contraction observed with all membranes?
5. Authors claim that the effective approach to realize WOW is inspired by skin formation, because in both cases, responsive macromolecular interactions are involved at liquid interfaces. Though it is valid to make the claim that biology makes use of water-water interfaces instead of oil-water interfaces, it is not fully clear to me, how the skin formation inspires the design of PEI-PSS on-water spreading? It is suggested to add the pH of PEI-PSS solution in Fig. 1a.
6. Though it is true that the surface spreading of aqueous polymer solution on water is rare, the two phases aqueous system driven by immiscibility of polymers should be discussed. More analysis in the differences between these two phenomena should be added for stressing the focal point of this work, especially to readers with interdisciplinary interests.
7. When the PSS-PEI solution was dropped on water, would a part of polyelectrolytes diffuse into water, instead of forming complexes. What is the polymer retention before and after surface spreading?
8. An advantage of this work is the facile procedure of experiments, and large size materials prepared. Is it true that the size of membrane is dependent in the volume of polyelectrolyte solution droplet? What is the biggest membrane size one can achieve by this method?

Reviewer #1 (Remarks to the Author):

In the manuscript entitled "Spontaneous, water-on-water spreading and assembly of polyelectrolytes inspired by skin formation", the authors proposed a water on water spreading method to construct porous polyelectrolyte films based on the complexation induced solidification mechanism. The spreading process, the influence of surface tension difference and pH on the spreading diameter, and the versatility of this method salt-triggered and solvent exchange-triggered film formation system were investigated. Finally, the authors also showed the application of this method to prepare CNT loaded porous film for solar evaporation. This work is interesting with a relatively clear and systematic logic. However, I still have some reservations, on addressing which the article can be improved further.

1. The characterization of the porous film. Only surface morphology of the prepared films is provided without the indication whether the characterized surface is the bottom interface contacting with water or the top surface contacting with air. Both surfaces should be characterized. The cross-sectional characterization of the porous film is suggested. In addition, the characterization in the thickness direction should also be provided to indicate the film uniformity.

Response: Thanks a lot for this good question. In this revision, we added Cryo-SEM characterizations of both the bottom (contacting with water) and top (contacting with air) surfaces of all PPMs and PPCMs.

As seen from Fig. R1 (top row), top surfaces of all PPMs are rougher and more porous compared to bottom surfaces which are smoother and less porous (bottom row). The asymmetric pore structures of PPMs are consistent with membranes prepared by non-solvents induced phase separation[R1] or aqueous phase separation[R2], in which surfaces contacting with non-solvents (analogues to water in this work) are normally denser. Moreover, pore sizes of PPMs increase with decreasing the pH of water. The PEI-PSS complexation is weaker and the spreading is quicker at higher pH, accompanied with membrane contraction as discussed before. In this scenario, the complexation is not strong enough to effectively solidify phase separated pores, and the back flow of PEI-PSSNa solution during the membrane contraction will fill in the pores formed during the contraction. As such, pore structures in PPMx (*x:* 2.25, 2.0) are less developed. Similarly, the top surface of PPCMs (Fig. R2, top row) are also more porous than that of the bottom surface, and pores are more developed at lower pH.

Reference:

[1] *Ind. Eng. Chem. Res.* **50**, 3798-3817 (2011).

[2] *Adv. Funct. Mater.* **30**, 1907344 (2020).

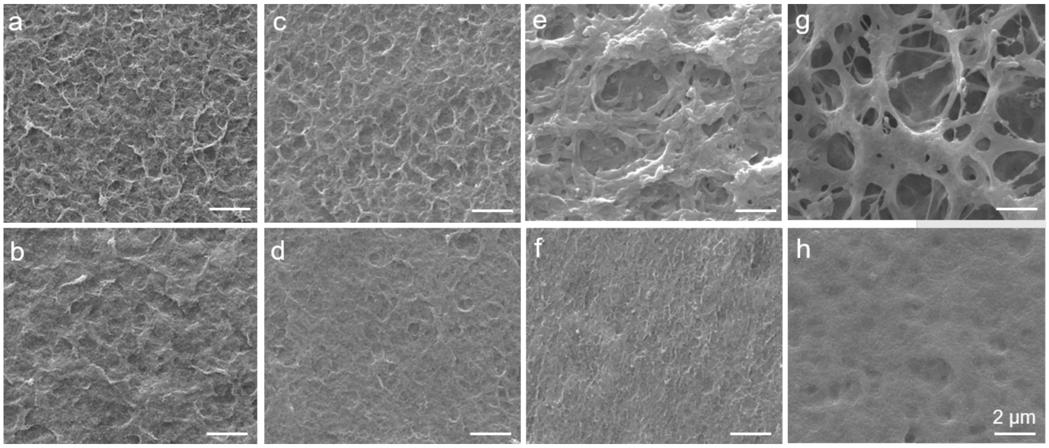

Fig. R1. Cryo-SEM images of (a,b) PPM2.25, (c,d) PPM2, (e,f) PPM1.75, and (g,h) PPM1.5. Please note: all scale bars are 2 μm. Images at top and bottom rows represent top (contacting with air) and bottom (contacting with water) surfaces of PPMs.

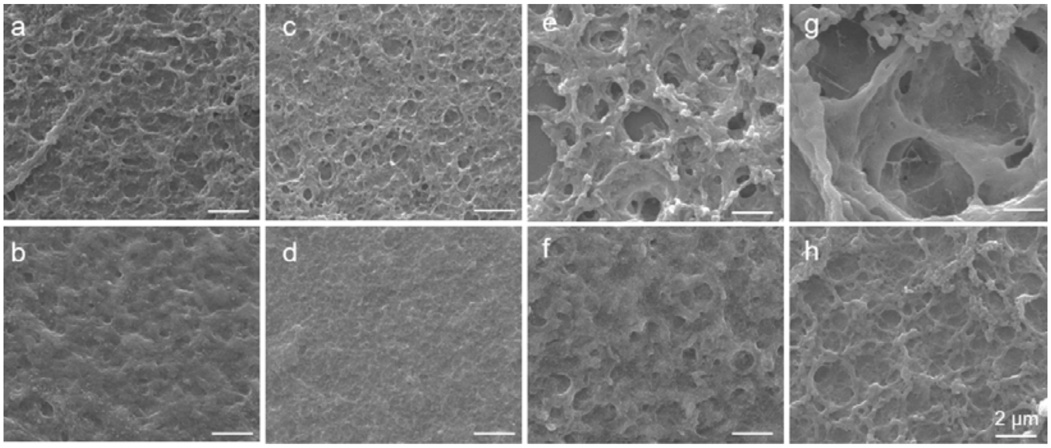

Fig. R2 Cryo-SEM images of (a,b) PPCM2.25, (c,d) PPCM2, (e,f) PPCM1.75, (g,h) PPCM1.5. Please note: all scale bars are 2 μm. Images at top and bottom row represent top (contacting with air) and bottom (contacting with water) surfaces of PPCMs.

Revisions made: Fig. R1 was added as Fig. 3. Fig. R2 was added supplementary Fig. S12.

Page 8, Lines 1-12: "Fig. 3 shows the Cryo-SEM characterizations of PPMs. As seen from Fig. 3 (top row), top surfaces (contacting with air) of all PPMs are rougher and more porous compared to bottom surfaces (contacting with water) which are smoother and less porous (middle row). The asymmetric pore structures of PPMs are consistent with membranes prepared by non-solvents induced phase separation or aqueous phase separation, in which surfaces contacting with non-solvents (analogues to water in this work) are denser. Moreover, pore sizes of PPMs increase with decreasing the pH of water. The PEI-PSS complexation is weaker and the spreading is quicker at higher pH, accompanied with membrane contraction as discussed before. In this scenario, the complexation is not strong enough to effectively solidify phase separated pores, and the back flow of PEI-PSSNa solution during the membrane contraction will fill in the pores formed during the contraction. As such, pore structures in PPMx (*x:* 2.25, 2.0) are less developed."

2. The formation mechanism focuses only on the diameter. The thickness or thickness profile change, i.e., the complexation mechanism in the thickness direction, with the variation of pH or should also be investigated. Will the membrane gradually contracting process influence the thickness uniformity?

Response: Thanks for this good question. Thickness of PPM2.25 along its diameter direction, and average thickness of different PPMs were measured by AFM (Fig. R3). We also added thickness-dependent XPS measurement of the PPM2.25, whereby the membrane was etched by argon beam for different times.

Putting together, we prepared a new Figure to enhance our discussion. Please see below.

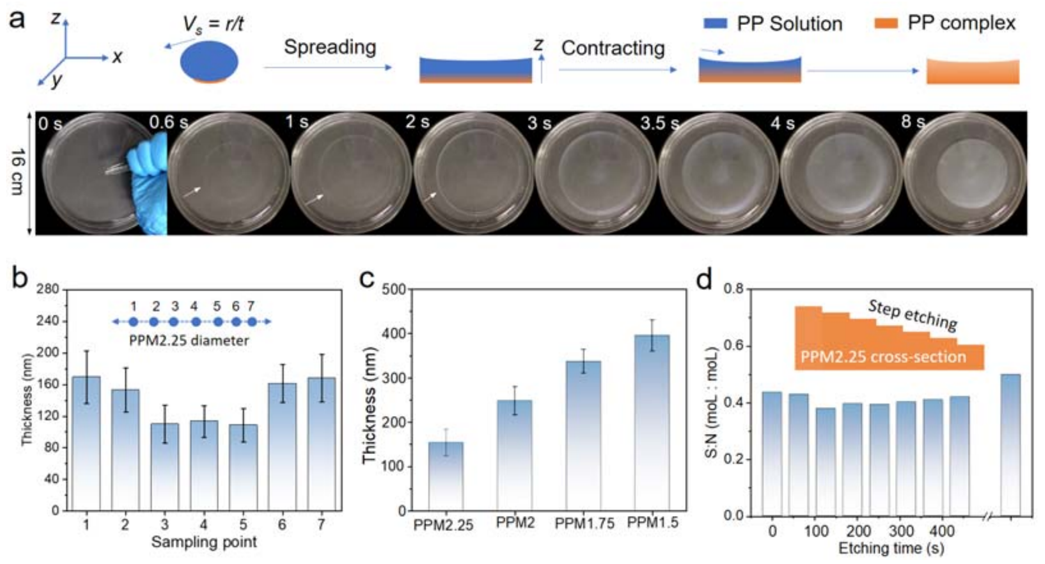

Fig. R3 (a) Optical photographs of the spreading of a PEI-PSSNa droplet on pH 2.25 water; inner diameter of the glass vessel is 16 cm. Schemes are inserted atop to illustrate the cross-sectional changes of the droplet. (b) Thickness of PPM2.25 at seven different locations along the diameter direction, (c) Average thickness of PPMs prepared by SWOW on water with different pH. (d) Mole ratio of sulfur and nitrogen elements at different depth down the PPM2.25 cross-section. Error bars: standard deviation.

Revisions made: Fig. R3 was added as Fig. 2 in the main text.

Page 6: "The contraction of PPM2.25 was further studied. As shown by arrows in Fig. 2a, the expanding periphery of PPM2.25 turns opaque quickly in the first 2 s of droplet spreading, while the rest of PPM2.25 remains transparent (Supplementary Video S1). This opaque periphery represents outer borderline of the spreading droplet, whereby the PEI-PSSNa complexation occurs since the beginning of the spreading process. With increasing the spreading time, this periphery became more and more solidified, which has a self-limiting effect in the droplet spreading. That is, the spreading solution eventually "bounce back" against the solidified periphery, resulting in the back flow of PEI-PSSNa solution accompanied with the contraction of PPM2.25. Meanwhile, as indicated by the change of orange color of the cross-sectional schemes in Fig. 2a, the complexation at thickness (z axis) direction started from the bottom surface contacting

with acidic water, and progressed toward the membrane's top surface contacting with air. The PEI-PSSNa complexation would result in phase separation, which was verified by the membrane's increasing opaqueness with increasing time (> 3 s)."

Page 6: "Thickness of air-dried PPM2.25 was measured along the diameter direction. Fig. 2b shows that the PPM2.25 is thicker (~ 160 nm) at its periphery part, thinner at the central part (~ 120 nm). This thickness profile is consistent with the membrane contraction of PPM2.25, *i.e.,* the back flow of PEI-PSSNa solution renders higher thickness at the membrane periphery. Fig. 2c shows that the average thickness of PPMs decreases with increasing pH of water. The PEI-PSS complexation is stronger at lower pH, leading to smaller D_{max} and higher thickness of PPMs. As shown by the inserted scheme in Fig. 2d, PPM2.25 was etched stepwise using argon-ions and measured by XPS. The mole ratio of sulfur element to nitrogen element is stable (~ 0.45) at different depth down the PPM2.25, and the sodium content throughout the membrane cross-section is very low (~ 0.1 wt%). These results suggest that both the complexation and chemical composition at thickness direction is homogeneous."

3. Coffee ring-like structure occurs in the CNT loaded film which leads to an uneven film. Does this phenomenon occur only in the CNT loaded the system or also in the CNT absent system? Is this related with the membrane gradually contracts process?

Response: Thanks a lot for this good question. This also occurs with CNT absent systems. Please see below one of the representative SEM images of PPM1.5 (Fig. R4). We believe the coffee ring structures are related to the Marangoni convection at the thickness direction during the surface spreading process. In literature, it has been known that Marangoni convection leads to coffee ring structures.

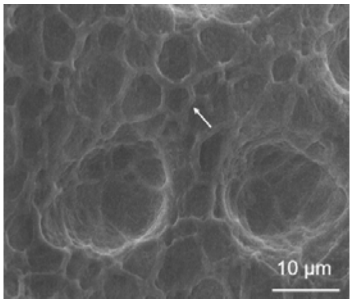

Fig. R4. SEM images of PPM1.5. Please note: the membrane was freezing dried and the arrow indicates coffee ring structures.

Reviewer #2 (Remarks to the Author):

The authors prepared polyelectrolyte complex membranes in acidic water using PSS and PEI and studied the solar thermal evaporation performance. The resultant polyelectrolyte complex membranes are well characterized. The methodology is sound and can be reproduced. The article has some typos which need to be corrected.

Response: Thanks a lot for the positive comments. Please see below Typos corrected.

"centries" was changed into "centuries"

"instuments" was replaced by "instruments"

The typo "excellular" and "exctracellular" were deleted
"occured" was changed into "occurred"
"a characteristic FT-IR peaks" was changed into "a characteristic FT-IR peak"
"was moved to" was changed into "shifts to"

The authors have claimed that they are the first to engineer such porous membranes, which is not true. There is a report in the literature that has studied the exact same polyelectrolytes for porous membranes production and has fine-tuned their properties. The reference to their work is missing (J. Mem. Sci. 2021, 625, 11911).

Response: Thanks for the suggestion. It was an inappropriate expression. We wrote in the original submission that "we proposed a fundamentally new mechanism which enables the SWOW and modulated engineering of porous PEC membranes for the first time." By this expression, authors meant to stress that the *water-on-water* spreading phenomenon is new. We DO NOT mean to claim that the polyelectrolytes are new. The expression was corrected and the reference was added. Please see below.

Revisions made: Page 15, Lines 11-12: a mechanism was proposed to enable the SWOW of regular-size water droplet, without the need for specialized equipment.

The suggested reference was added as Ref 36, cited in four places (Page 3, Lines 12; Page 4, Line 7; Page 8, Line 17 and Line 20.

Furthermore, the authors have only 'mentioned' the results without providing sufficient reasoning. For example, why does the film diameter increase in the beginning and then decrease? Why does the PPM2.25 membrane have smaller pores as compared to PPCM1.5 membranes even though the latter was prepared at lower pH values? How does the addition of CNT affect the membrane formation kinetics and why does it retain the higher pore size? How does CNT attach to the polyelectrolyte complex? The discussion section severely lacks such reasoning and is only a summary at the moment.

Response: Thanks for this good question. Please see response below.

Membrane contraction. This question was also asked by Reviewer#1. In general, the membrane contraction is due to the self-limiting effect of PEI-PSS complexation in the spreading process. That is, PEI-PSS solution "bounces back" against the solidified periphery of PPMs during the spreading. Please see revisions below.

Revision made: Relevant data (Fig. 2 in the main text) and discussions can be found in Page 6, Lines 7-19, and Page 7.

Membrane pores. We measured both the top and bottom surfaces of PPMs and PPCMs by Cryo-SEM. In general, the top surfaces are more porous than the bottom surface. Moreover, average pore sizes increase with decreasing pH, whereby the phase separation is more rapid.

Revisions made: Relevant data (Fig. 3 in the main text) and discussions can be found in Page 8, Lines 1-12, and Page 9.

Effect of CNTs in SWOW. The zeta potential of CNT dispersion is ca. -10 mV, indicating that CNTs bear moderate negative charge, which is reasonable because CNTs used in this work were NOT treated by strong acid. The negative charge could enhance

its interaction with PEI. As shown in Fig. R5, adding CNTs in the PEI-PSSNa solution does not significantly change the membrane diameter of PPCMs. The spreading speed PPCM was slightly reduced when the CNT content is as high as 20 wt%.

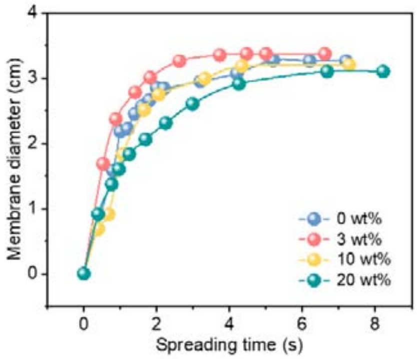

Fig. R5. Dependence of membrane diameter on the CNT content in the PEI-PSSNa droplet. Please note: the droplet was spread on pH 1.5 water.

Revisions made: Fig. R5 was added as Supplementary Fig. 15.

Page 12, Lines 13-15. “It is worth mentioning that zeta potential of CNTs dispersion (0.1 mg/L) is ca. -10 mv, indicating that CNTs bear moderate negative charge which strengthens their interaction with PEI polymers.”

Page 12, Lines 18-21. “Compared to PPMs, the addition of moderate amount of CNTs does not significantly change the SWOW spreading of PEI-PSSNa-CNT mixture, in terms of spreading speed, membrane diameter and pore structures (Supplementary Fig. 15).”

Therefore, in light of these comments, I cannot recommend the publication of this article in Nature communications in its current form.

Reponses: Thanks to your enlightening suggestion, , we have substantially improved the manuscript in this revision. The suggested references represent nice contributions focusing on the phase inversion polyelectrolyte complex membranes prepared by immersion-induced-complexation, which could supplement the current NIPS method in membrane industry. Actually we have also investigated porous poly(ionic liquid) complex membrane by somehow similar strategy in previous studies. This work, though use similar polyelectrolyte, have a different focus on the surface spreading phenomena.

Specific Comments:

1. The authors should mention in the introduction why PSS and PEI were specifically chosen for this application?

Response: Thanks a lot for this good question. The aim of this work is to engineer *water-on-water* surface spreading, contrast to the classic *oil-on-water* spreading. PSS solution was chosen due to its lower surface tension (60 mN/m), which provides the driving force for surface spreading. Meanwhile, PEI was chosen to form a homogeneous solution with PSS, which undergo pH-responsive complexation.

Revisions made, Page 3, lines 13-15. “In this design, the PSSNa endows the mixture

solution with surface tension lower than water, which is the driving force for surface spreading. Meanwhile, PEI is a weak polyelectrolyte bearing positive charge at acid condition, which renders pH-responsive complexation [34-36]."

2. Porous PSS-PEI membranes are already reported in the literature. The authors have followed a similar protocol for the preparation of polymer solutions and subsequent precipitation. Please add the relevant reference.

Response: Thanks a lot. The suggested references were added. Noteworthy, the principle for SWOW applies not only to the PSS-PEI, but also to other systems such as PDDA-PSS and PCMVImTf$_2$N-PAA (Supplementary Fig. 10, Fig. 11). In the latter two systems, the complexation was screened first by salts or organic solvents, and was then triggered by "salt-diluting" or "carboxyl de-pronation", respectively. Previously, we have used this principle to prepare porous poly(ionic liquid) actuators [Nat. Commun. 2014, 5, 4293] and wet adhesives [Nat. Mater. 2016, 15, 407].

Revisions made, Page 4, Lines 10-12. "The principle to prepare homogeneous polyelectrolytes mixture is to first screen the charge attraction between different polyelectrolytes, which is consistent with previous reports [37-39]."

[37] *ACS Appl. Polym. Mater.* **2**, 2612-2621 (2020).

[38] *ACS Appl. Mater. Inter.* **11**, 16018-16026 (2019).

[39] *Nat. Mater.* **15**, 407-412 (2016).

3. Line 118. The word 'mini-seconds' is not scientific and should be removed. The ref 36 which it corresponds to studies sodium polyacrylate (SPA) and polyallylamine hydrochloride (PAH) complexation as opposed to PSS-PEI studied in this work. The kinetics are very different for these complexes and are a function of several variables including the pH of the solution and water bath. Reference on PSS-PEI complexation and its kinetics would be more accurate.

Response. Thanks a lot for this good question. It is widely recognized that the kinetics of polyelectrolyte complexation is rapid, though the detailed kinetics would vary depending on the complexation systems. As suggested by the reviewer, the PEI-PSS complexation kinetics is also influenced by the protonation of NH_2 groups. According to the reviewer's comment, we added two references which studied the PEI-PSSNa complexation by immersing the solution in coagulation bath. Please see below.

Revision made. Page 8, Lines 16-17: "Consequently, the protonated PEI undergo concurrent PEI-PSSNa complexation that occurs rapidly [36]."

[36] *J. Membr. Sci.* **625**, 119114 (2021).

4. The statement on page 6 line 121 is misleading. The polyelectrolyte complexation on liquid-liquid interfaces has been studied by several groups and there are numerous articles on this topic. (ACS Appl. Mater. Interfaces 2019, 11, 17, 16018–16026) (ACS Appl. Polym. Mater. 2020, 2, 7, 2612–2621) (J. Mem. Sci. 2021, 625, 11911).

Response: Thanks for this good question. We regret for the misleading expression which was due to the inappropriate use of language. In the original submission, we

mentioned that “polyelectrolyte complexation mainly occurred in solution….”, by the expression “complexation in solution”, we intended to mean complexation that occurs in the bulk of aqueous solution, instead of surface spreading. We have corrected this clumsy expression. Please see below.

Page 8, lines 20-22. Polyelectrolyte complexation mainly occurred at liquid-liquid [36-38] and/or liquid-solid interfaces[45,46]. Here, the polyelectrolyte complexation is coupled to the surface spreading of aqueous solution on water.

Reference added:

[36] *J. Membr. Sci.* **625**, 119114 (2021).

[37] *ACS Appl. Polym. Mater.* **2**, 2612-2621 (2020).

[38] *ACS Appl. Mater. Inter.* **11**, 16018-16026 (2019).

5. Why pH 1.5 for CNT and why 2.25 for PP? The authors said that reducing pH results in stronger complexation. Then why the pores are larger for 1.5 than 2.25? The authors correlate this to freeze-drying process which is unfair. Here, complexation is the main mechanism and the authors need to verify the effect of CNT on the PSS-PEI complexation. Could it be that the CNTs are charged and interfering in the complexation? More elaborate reasoning is required.

Response: Thanks a lot. PPM2.5 shows the largest membrane diameter, which is suited for the study of SWOW phenomenon. PPCM1.5 was relatively thicker, beneficial for light absorption and water evaporation. In this revision, we characterized morphologies of all PPMs and PPCMs by Cryo-SEM (Fig. R1, R2). Please find detailed discussion in Fig. 2 in the main text (Page 8, Page 9).

According to the reviewer’s suggestion, we deleted the correlation between “freezing drying” process and the current process. The reviewer is true that CNTs used in this work are moderately charged (zeta potential: ca. -10 mV). Yet the CNTs’ charge is not very strong, and its interference in polyelectrolyte complexation is not significant, as seen by the effect of CNT in SWOW (Fig. R5).

Revisions made. Fig. R5 was added as Supplementary Fig. 15. Relevant discussion can be found (Page 12, Lines 13-15.) and Page 12, Lines 18-21.

Page 12, Line 7: “PPCM1.5 with relatively larger thickness was chosen for photothermal evaporation study.”

The following expression was deleted: “In literature, linear fillers are reported to stabilize pores during freezing drying process”.

Reviewer #3 (Remarks to the Author):

Tang et al. present a simple and efficient method of making thin polyelectrolyte films by interfacial precipitation of spreading droplets. The formation of membranes by interfacial precipitation is well known, but the mechanism described in this paper includes in a novel way spontaneous, water-on-water spreading (SWOW) to form membranes with hierarchical pores (claimed to be thinner and more uniform compared to other membranes made by bulk precipitation). The resulting hierarchical porosity is

interesting and potentially quite useful. The method is much faster, though likely less precise than the common polyelectrolyte layer-by-layer deposition. The material is overall publishable in a journal of this magnitude, but presently has a number of drawbacks that need to be addressed before a decision of publication can be made. These drawbacks include:

1. The authors present data on membrane diameter, but do not present the other critical data on membrane thickness and thickness inhomogeneity. The thickness is obviously larger at the periphery, and the membranes show significant wrinkling. The control over the thickness and uniformity of the films should be presented with data and analysis.

Response: Thanks a lot. The reviewer is correct that the thickness is relatively larger at the periphery of PPM2.25, which is likely due to the membrane contract. In addition, the thickness of PPMs is inversely correlated to the pH of underlying water, which is an effective means of thickness modulation. Similar questions have been asked by other reviewers. Please see revisions below.

Revisions made: Fig. R3 was added as Fig. 2 in the main text (Page 6, Page 7).

2. The authors use the values of the static equilibrium surface tension to describe the process of dynamic spreading, during which the interfacial tensions can vary and approach the equilibrium one towards the end. This difference and the assumptions used in data interpretation should be explained carefully.

Response: Thanks a lot. We agree that the interfacial tensions can vary during the spreading. Surface tension of underlying water (after PEI-PSSNa spreading) is measured to be 74.5 ± 0.5 mN/m, close to the surface tension of water. Revisions were made According to the reviewer's suggestions.

Revisions made: Page 9, Lines 10-13. "Noteworthy, the value of γ_w would vary slightly during the spreading of PEI-PSSNa solution, and the γ_w is measured to be 74 ± 0.5 mN/m (> γ_s) when the spreading is finished. Here, the static γ_w, instead of dynamic γ_w, was used to qualitatively analyze the effect of $\Delta\gamma$ in the maximum diameter of PPMs (*eq. 1*)."

3. The data for photothermal evaporation are interesting, but unclearly described. The major parameter for comparison between the PPCM and PPM films should be the amount of CNTs deposited per unit area, which is hard to estimate. Only then one can make a conclusion whether the special porosity contributes to the more effective light capture as claimed in the paper. Also, the authors should provide more information of how CNT affects mechanical properties of the membrane (assuming they may act as structural fillers).

Response: Thanks a lot for this good question. Dense PPMs and PPCMs (without pores, 3 wt% CNT) were prepared. As shown in Fig. R6a in this file, the light absorption of dense PPM and dense PPCM are lower than that of PPM and PPCM, respectively. Fig. R6b shows that the both the temperature and water evaporation rate of PPCM1.5 and PPM1.5 are higher than that of dense PPCM1.5 and dense PPM1.5, respectively. These results verify our speculation that the porous structure enhances light absorption

through the light reflection on pore walls, which confers higher water evaporation rate to PPCMs. Last, it is qualitatively seen that the strength of PPCM is higher than that of PPM, yet both membranes are too thin to show enough strength for quantitative measurements. Furthermore, we found that the incorporation of CNTs does not obviously change the D_{max} of the PPCMs (Fig. R5).

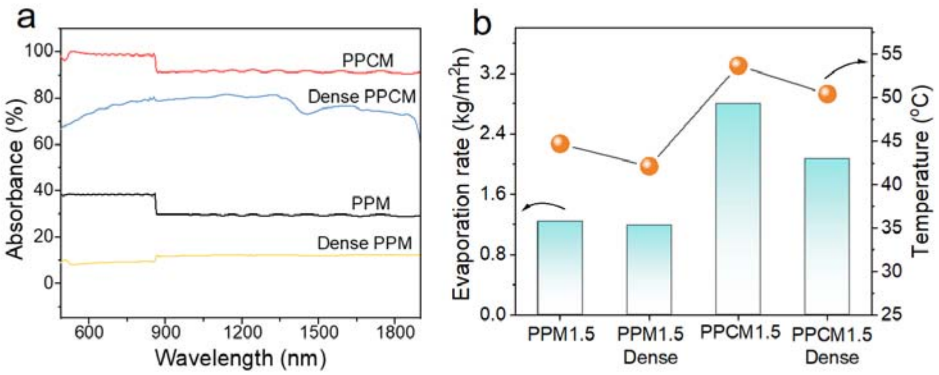

Fig. R6 (a) UV-vis absorption and (b) water evaporation rate (1-sun irradiation) of PPM1.5, dense PPM1.5, PPCM1.5, and dense PPCM1.5. Please note: dense PPMs were prepared by casting the PEI-PSSNa (or PEI-PSSNa-CNT) solution on glass plates, following by drying (50 °C, 2 h) and acid annealing.

Revisions made: Fig. R6 was added as Supplementary Fig. 17.

Page 13, Lines 10-13: "Indeed, it is found that the light absorption of a control membrane, *i.e.,* a dense PPM contains a similar amount of CNT, shows lower light absorption temperature under 1-sun irradiation (Supplementary Fig. 17)."

4. The paper is in some places written in hard to read style, and contains unclear sentences, buzzword phrases, trivialities, and some exaggerations. This is especially problematic for the discussion section. The whole paper should be revised carefully to match the requirements for clarity and precise expression expected for a journal of such magnitude.

Response: Thanks a lot for this question. We have revised the manuscript carefully to present the work in a clear way, with particular attention paid to the discussions. These revisions can be found in Page 15 and Page 16 in the main text.

5. The method suffers from some disadvantages, namely the inability to make membranes of arbitrary shapes, disruption by potential interfacial instabilities, uneven thickness at periphery, difficulty in adapting to continuous film withdrawal. These disadvantages are not critical, but should be mentioned clearly, presenting the method in a balanced manner.

Response: Thanks a lot for this question. The challenges, e.g., making membrane with arbitrary shapes, are of interests for future research. According to the reviewer's suggestion, please see below the discussions we added.

Revision made: Page 16, Lines 20-24. "For example, it remains unclear how to prepare membranes with arbitrary shapes using the SWOW mechanism. In addition, the surface spreading of polyelectrolyte solution is a self-limiting process, *i.e.,* polyelectrolytes

were solidified quickly, due to which the continuous withdrawal of SWOW membranes is challenging."

Reviewer #4 (Remarks to the Author):

Liquids interface is interdisciplinary topics widely important for chemistry, materials and engineering. This work represents a concrete step beyond the conventional context of oil-water spreading, and manages water-water spreading and materials functionality ingeniously. Authors stabilized miscible interfaces by pH responsive polyelectrolyte complexation featuring quick kinetics. The entire experiments were conducted with rational procedures, and the formation mechanism of the porous membranes has also been well demonstrated. Importantly, this work provided an inspiring approach to tackle the tricky water-water interfaces that quickly mix. Authors rationally propose and verify the mechanism of the WOW spreading. They also demonstrated the implication of WOW spreading for scalable preparing porous membranes with excellent solar thermal evaporation. The strength of this work lies in the WOW mechanism underpinning wide implications in chemistry and materials. Given the broad interest and high standard of nature communications, I recommend its publication in this journal pending revisions beneath.

1.The responsive PSS-PEI complexation is the key to support WOW spreading. Authors tried to prove the complexation by FT-IR which indicates the protonation and deprotonation of amine groups. Though this data is useful, could authors provide complementary data to support electrostatic complexation in a more straightforward manner?

Response: Thanks a lot for the positive comments. The FT-IR shows the charge state of amine groups, *i.e.,* NH_2 and NH_3^+, and verifies the complexation. In addition, ICP characterization of PPM2.25 was added, showing that the membrane contains negligible amount of Na^+ (~0.1 wt%**)**. As schemed in Fig. R7, the low amount of Na^+ in PPM is due to the PEI-PSSNa complexation, during which the Na^+ ions in PSSNa were released as free ions due to the complexation. This is a direct proof of PEI-PSSNa complexation. Similar methods have been used to prove polyelectrolyte complexation [Nat. Mater. 2016, 15, 407–412].

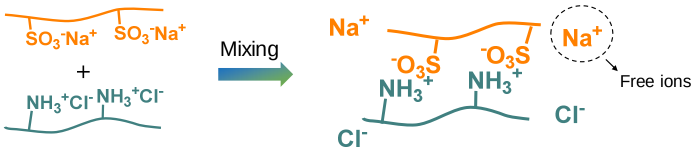

Fig. R7. Schematic illustration of the release of Na^+ counter-ions as a result of PEI-PSS complexation.

Revision made: Fig. R7 was added as Supplementary Fig. 4.

Page 6, Lines 2-6 "Finally, the content of Na^+ in the PPM2.25 is very low (~ 0.1 wt%), while the Na^+ content in PSSNa is calculated to be ~ 12 wt%. This is a direct proof of the PEI-PSSNa electrostatic complexation, during which the Na^+ counter ions of PSSNa were released as free ions and removed during the membrane washing

(Supplementary Fig. 4)."

2. Gravity of the solution droplet also plays a role in spreading. What is the density of the PSS-PEI solution, higher or lower than water?

Response: Thanks. The density of PSS-PEI solution is 1.04 g/mL, which is close to that of water. This information was added in the experimental part.

3. Authors proposed two sets of control experiments (Fig. 3) to support the WOW mechanism. While the mechanism is convincing in general, the reviewer is curious to know the effect of more factors in the WOW process, such as adding surfactants that could be easily imagined?

Response: Thanks a lot. According to the reviewer's suggestion, surfactants (sodium dodecyl sulfonate) were added in the water. Fig. R8a shows that the $\Delta\gamma$ was reduced with increasing the amount of surfactants in water. Reasonably, the driven force of the spreading was reduced, leading to smaller D_{max} of PPMs (Fig. R8b). This observation is consistent with the proposed mechanism of SWOW.

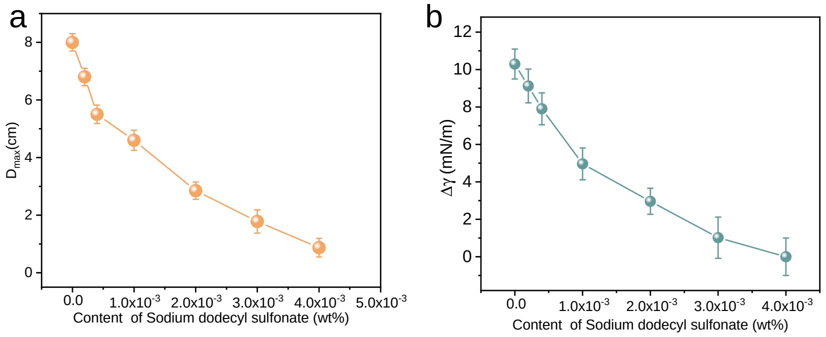

Fig. R8. Effect of sodium dodecyl sulfonate content in (a) D_{max} and (b) $\Delta\gamma$ of PPM1.75. Error bars: standard deviation.

Revision made. Fig. R8 was added as Supplementary Fig. 9.

Page 11, Lines 5-7. "In addition, similar "$D_{max} \sim \Delta\gamma$" correlation was observed when γ_w was tuned by adding surfactants in water (Supplementary Fig. 9)."

4. Authors mentioned the membrane contraction in the second stage of spreading. What is the reason for membrane contraction? Is the contraction observed with all membranes?

Response: Thanks a lot for this question. The membrane contraction is observed with PPM2.25, PPM2.0, and PPM1.75, while the contraction is trivial for PPM1.5. The reason of membrane contraction is due to the "bouncing back" of PEI-PSS solution against the solidified periphery of PPMs during the spreading. Similar question was asked by other reviewers, and a new Figure (Fig. R3) was added to discuss this.

Revision made: Fig. R3 was added as Fig. 2 in the main text. Please see related discussion (Page 6, Page 7)

5. Authors claim that the effective approach to realize WOW is inspired by skin formation, because in both cases, responsive macromolecular interactions are involved

at liquid interfaces. Though it is valid to make the claim that biology makes use of water-water interfaces instead of oil-water interfaces, it is not fully clear to me, how the skin formation inspires the design of PEI-PSS on-water spreading? It is suggested to add the pH of PEI-PSS solution in Fig. 1a.

Response: Thanks a lot. As suggested by the reviewer, the pH of PEI-PSS solution was added in Fig. 1a. Normally, two aqueous solutions would mix with each other. In biology, by contrast, phase changes of protein mixtures would be triggered by environmental stimulus such as pH changes. Take the skin barrier formation for example, phase-transitions of protein granules was triggered by the pH changes during its upward journey toward the skin surface. We are inspired by this mechanism, and exploited pH-dependent PSSNa-PEI complexation to stabilize water-water interfaces.

Revisions made: Page 3, Lines 2-4: ", which hints that stimuli-dependent interactions between water-soluble polymers might be viable for stabilizing "water ~ water" dynamic interfaces between two aqueous polymer solutions."

6. Though it is true that the surface spreading of aqueous polymer solution on water is rare, the two phases aqueous system driven by immiscibility of polymers should be discussed. More analysis in the differences between these two phenomena should be added for stressing the focal point of this work, especially to readers with interdisciplinary interests.

Response: Thanks a lot for this question. The two phases aqueous system normally occurred in the bulk of aqueous polymer solution, not at the air-water surface. We have stressed the difference of the two processes. Please see revisions beneath.

Revisions made: Page 4, Lines 20-21: "In literature, "water ~ water" interfaces can exist in the bulk of two-phases aqueous systems[21], which are not viable for *water-on-water* surface spreading."

7. When the PSS-PEI solution was dropped on water, would a part of polyelectrolytes diffuse into water, instead of forming complexes. What is the polymer retention before and after surface spreading?

Response: Thanks a lot for this question. Taking the PPM1.5 for example, the mass retention before and after surface spreading is ca. 75 wt%. The loss of mass includes counter ions and a part of polymers that diffused into water before they are solidified by complexation. Please see revisions below.

Revision made: Page 17, Lines 12-13: "Taking PPM1.5 for example, the mass retention of PEI and PSS polymers is ~ 75%."

8. An advantage of this work is the facile procedure of experiments, and large size materials prepared. Is it true that the size of membrane is dependent in the volume of polyelectrolyte solution droplet? What is the biggest membrane size one can achieve by this method?

Response: Thanks for this question. It is true that the size of membranes depends on the volume of the polymer solution droplet. When a droplet with a larger volume (e.g., 140 μL) was placed on water (pH 2.25), the D_{max} is ca. 18 cm (Fig. R9).

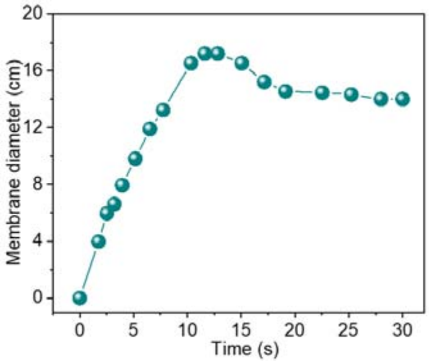

Fig. R9 Effect of spreading time on membrane diameter. Please note: the volume of PEI-PSSNa droplet is 140 μL.

Revision made: Fig. R9 was added as Supplementary Fig. 3.

Page 4, Lines 16-17. ", which also depend on the volume of the droplet (Supplementary Fig. 3)."

Reviewers' Comments:

Reviewer #1:
Remarks to the Author:
The authors have answered most of our comments. The authors misunderstood the “coffee ring” structure we mentioned, which is the coffee ring structure in Fig. 5a of the revised manuscript, where the film center is thinner and the periphery is thicker. As the authors have proved that the film thickness is not homogeneous while the complexation and chemical composition at thickness direction are homogeneous, it is acceptable for publication.

Reviewer #2:
Remarks to the Author:
The revised manuscript provides a clear understanding of SWOW using the polyelectrolytes studied. The authors have done a great job in revising the manuscript. The results are properly explained with relevant characterization and all the reviewer's comments have been well addressed in the revised version. The quality of the manuscript is now significantly enhanced. This article certainly contributes toward the broader scientific understanding of SWOW using polyelectrolytes. I appreciate the authors' work on the revised manuscript.

Reviewer #3:
Remarks to the Author:
I have examined the revised manuscript and the responses to the reviewers. It is interesting to note that there is very good correspondence between the generally positive impressions of all 4 reviewers and many of the issues raised by the reviewers are overlapping. Overall, I think the authors have addressed the reviewers' comments thoroughly and the additions/edits that they've made to the manuscript will help clarify most of the questions.

The only comment I did not find addressed in depth form my comments was the change of the mechanical properties of the membranes following the addition of CNTs. The authors qualitatively state that this strengthens it. There could be a number of ways to evaluate whether the strength and other mechanical properties change even if semi-quantitatively, such as also checking whether they can be bent, creased, challenged with small weights, etc. However, this is a minor issue, and given the very large number of other data sets presented the paper can be accepted as is, unless more serious issues have been raised by other reviewers and demand another revision.

Reviewer #4:
Remarks to the Author:
The authors have addressed all my concerns, this reviewer recommended to publish this work on NC.
